# Vigorous Intermittent Lifestyle Physical Activity and Cancer Incidence Among Nonexercising Adults: The UK Biobank Accelerometry Study

**DOI:** 10.1001/jamaoncol.2023.1830

**Published:** 2023-07-27

**Authors:** Emmanuel Stamatakis, Matthew N. Ahmadi, Christine M. Friedenreich, Joanna M. Blodgett, Annemarie Koster, Andreas Holtermann, Andrew Atkin, Vegar Rangul, Lauren B. Sherar, Armando Teixeira-Pinto, Ulf Ekelund, I-Min Lee, Mark Hamer

**Affiliations:** 1 Mackenzie Wearables Research Hub, Charles Perkins Centre, University of Sydney, Sydney, New South Wales, Australia; 2 School of Health Sciences, Faculty of Medicine and Health, University of Sydney, Sydney, New South Wales, Australia; 3 Department of Cancer Epidemiology and Prevention Research, Cancer Care Alberta, Alberta Health Services, Edmonton, Alberta, Canada; 4 Departments of Oncology and Community Health Sciences, Cumming School of Medicine, University of Calgary, Calgary, Alberta, Canada; 5 Institute Sport Exercise Health, Division Surgery Interventional Science, University College London, London, United Kingdom; 6 Department of Social Medicine, Care and Public Health Research Institute, Maastricht University, Maastricht, the Netherlands; 7 National Research Centre for the Working Environment, Copenhagen, Denmark; 8 School of Health Sciences and Norwich Epidemiology Centre, University of East Anglia, Norwich, United Kingdom; 9 HUNT Research Centre, Department of Public Health and Nursing, Norwegian University of Science and Technology, Levanger, Norway; 10 School of Sport Exercise and Health Sciences, Loughborough University, Loughborough, United Kingdom; 11 Department of Sport Science and Physical Education, University of Agder, Kristiansand, Norway; 12 School of Public Health, Faculty of Medicine and Health, The University of Sydney, Sydney, New South Wales, Australia; 13 Department of Sport Medicine, Norwegian School of Sport Sciences, Oslo, Norway; 14 Department of Chronic Diseases, Norwegian Public Health Institute, Oslo, Norway; 15 Division of Preventive Medicine, Brigham and Women’s Hospital, Harvard Medical School, Boston, Massachusetts, United States; 16 Department of Epidemiology, Harvard T. H. Chan School of Public Health, Boston, Massachusetts, United States

## Abstract

**Importance:**

Vigorous physical activity (VPA) is a time-efficient way to achieve recommended physical activity (PA) for cancer prevention, although structured longer bouts of VPA (via traditional exercise) are unappealing or inaccessible to many individuals.

**Objectives:**

To evaluate the dose-response association of device-measured daily vigorous intermittent lifestyle physical activity (VILPA) with incident cancer, and to estimate the minimal dose required for a risk reduction of 50% of the maximum reduction.

**Design, Setting, and Participants:**

This was a prospective cohort analysis of 22 398 self-reported nonexercising adults from the UK Biobank accelerometry subsample. Participants were followed up through October 30, 2021 (mortality and hospitalizations), or June 30, 2021 (cancer registrations).

**Exposures:**

Daily VILPA of up to 1 and up to 2 minutes, assessed by accelerometers worn on participants’ dominant wrist.

**Main Outcomes and Measures:**

Incidence of total cancer and PA-related cancer (a composite outcome of 13 cancer sites associated with low PA levels). Hazard ratios and 95% CIs were estimated using cubic splines adjusted for age, sex, education level, smoking status, alcohol consumption, sleep duration, fruit and vegetable consumption, parental cancer history, light- and moderate-intensity PA, and VPA from bouts of more than 1 or 2 minute(s), as appropriate.

**Results:**

The study sample comprised 22 398 participants (mean [SD] age, 62.0 [7.6] years; 10 122 [45.2%] men and 12 276 [54.8%] women; 21 509 [96.0%] White individuals). During a mean (SD) follow-up of 6.7 (1.2) years (149 650 person-years), 2356 total incident cancer events occurred, 1084 owing to PA-related cancer. Almost all (92.3%) of VILPA was accrued in bouts of up to 1 minute. Daily VILPA duration was associated with outcomes in a near-linear manner, with steeper dose-response curves for PA-related cancer than total cancer incidence. Compared with no VILPA, the median daily VILPA duration of bouts up to 1 minute (4.5 minutes per day) was associated with an HR of 0.80 (95% CI, 0.69-0.92) for total cancer and 0.69 (95% CI, 0.55-0.86) for PA-related cancer. The minimal dose was 3.4 minutes per day for total (HR, 0.83; 95% CI, 0.73-0.93) and 3.7 minutes for PA-related (HR, 0.72; 95% CI, 0.59-0.88) cancer incidence. Findings were similar for VILPA bout of up to 2 minutes.

**Conclusions and Relevance:**

The findings of this prospective cohort study indicate that small amounts of VILPA were associated with lower incident cancer risk. Daily VILPA may be a promising intervention for cancer prevention in populations not able or motivated to exercise in leisure time.

## Key Points

**Question:**

Does vigorous intermittent lifestyle physical activity (VILPA) in short bouts (≤1 and ≤2 minutes) have a dose-response association with incident cancer among nonexercising adults?

**Findings:**

In this prospective cohort study of 22 398 self-reported nonexercising adults, a minimum dose of 3.4 to 3.6 minutes of VILPA per day was associated with a 17% to 18% reduction in total incident cancer risk compared with no VILPA. A median daily VILPA of 4.5 minutes was associated with a 31% to 32% reduction in physical activity–related cancer incidence.

**Meaning:**

The findings of this large cohort study suggest that 3 to 4 minutes of VILPA per day may be associated with decreased cancer incidence risk; thus, VILPA may be a promising intervention for cancer prevention among individuals unable or unmotivated to exercise in leisure time.

## Teaser

This prospective cohort study evaluates the dose-response association of device-measured daily vigorous intermittent physical activity with incident cancer risk among a large cohort of inactive adults.

## Introduction

The association between physical activity (PA) intensity and certain cancer sites, such as breast and colon^1^ cancers, is dose dependent and has a greater risk reduction associated with vigorous physical activity (VPA) compared with lower intensities. Although VPA is time efficient, structured exercise bouts may not be feasible or appealing to most middle-aged adults.^2^ Vigorous intermittent lifestyle physical activity (VILPA)^3^ refers to brief and sporadic (eg, up to 1-2 minutes [min]) bouts of VPA during daily living, eg, bursts of very fast walking or stair climbing. Therefore, VILPA cannot be captured by questionnaires; wearable trackers are essential. A recent study^4^ found a beneficial association of daily VILPA with total cancer mortality, although the low number of cancer deaths precluded a detailed dose-response examination. To our knowledge, no study has evaluated the associations of VILPA with cancer incidence. In a large sample of inactive adults (nonexercisers) we assessed the dose-response associations of device-measured daily VILPA duration with incidence of cancer and estimated the minimum VILPA amounts for cancer risk reduction.

## Methods

Details of the sample selection and study methods are described^4^ in eFigure 1 and the eAppendix in Supplement 1. We used the UK Biobank wrist accelerometry substudy activity data,^5^ a study previously approved by the UK National Research Ethics Service (No. 11/NW/0382) that had obtained written informed consent from all participants. We followed the Strengthening the Reporting of Observational Studies in Epidemiology (STROBE) reporting guideline (eTable 5 in Supplement 1).

From the UK Biobank wrist accelerometry substudy activity data,^5^ we included only nonexercisers—participants who reported no leisure time exercise and 1 or fewer recreational walks per week^4^ (eTable 1 in Supplement 1). We excluded participants with missing covariates; prevalent cancer or previous cancer in remission; cancer event during the first year after accelerometry baseline; or inadequate wear time^5^ (eTable 2 in Supplement 1). Physical activity intensity was classified using a validated^4,6^ 2-stage machine learning-based Random Forest activity classifier covering VPA, moderate-intensity physical activity (MPA), and light-intensity physical activity (LPA; eAppendix in Supplement 1). We tested bouts of up to 1 or 2 min, based on recent data showing that the mean (SD) time required to reach vigorous intensity during 5 typical VILPA activities is 73.5 (26.2) seconds.^4^ Cancer incidence was defined as cancer registration, hospitalization for cancer, or death attributed to any cancer, and excluded in situ, nonmelanoma skin cancer, and non−well-defined cancers. We also derived a composite cancer outcome of 13 sites that have been shown to be associated with PA (eTable 3 in Supplement 1).^7^ Participants were followed up through October 30, 2021 (mortality and hospitalizations), or June 30, 2021 (cancer registrations).

We assessed dose response of adjusted absolute risk between daily VILPA duration and incident cancer using Poisson regression.^6^ Time-to-event associations of daily VILPA duration were analyzed using Fine-Gray subdistribution hazards that account for competing risks from non−outcome-related causes of death.^8^ Analyses were adjusted for age, sex, body mass index (calculated as weight in kilograms divided by height in meters squared), education level, smoking status, alcohol consumption, sleep duration,^9^ fruit and vegetable consumption, medications, parental cancer history, prevalent cardiovascular disease, daily durations of all LPA and MPA, and daily duration of longer VPA bouts, as appropriate (eTable 4 in Supplement 1). We estimated^4,6^ the minimal VILPA dose as that associated with 50% of the optimal risk reduction.^10^ We used evenly spaced knots at the 6th, 34th, and 67th percentiles to fit the right-skewed VILPA distribution; E-values estimated the plausibility of unmeasured confounding.^4,6^ The interpretation of data was based on 95% CIs across the dose response curves (not *P* value testing). Data analyses were performed February to March 2023 using R, version 4.2.1 (The R Foundation for Statistical Computing) with RMS, version 6.3.0 (Harrell FE) and Survival package, version 3.3.1 (RStudio).

## Results

The study sample comprised 22 398 participants (mean [SD] age, 62.0 [7.6] years; 10 122 [45.2%] men and 12 276 [54.8%] women; 303 [1.4%] Asian, 237 [1.1%] Black, 142 [0.6%] multiracial, 21 509 [96.0%] White, and 207 [0.9%] individuals of other race/ethnicity; eTable 6 in Supplement 1). During a mean (SD) follow-up of 6.7 (1.2) years (149 650 person-years), 2356 new cancer events occurred (1084 in PA-related cancer sites); 6.2% of the study participants recorded no VILPA.

**Figure 1.**
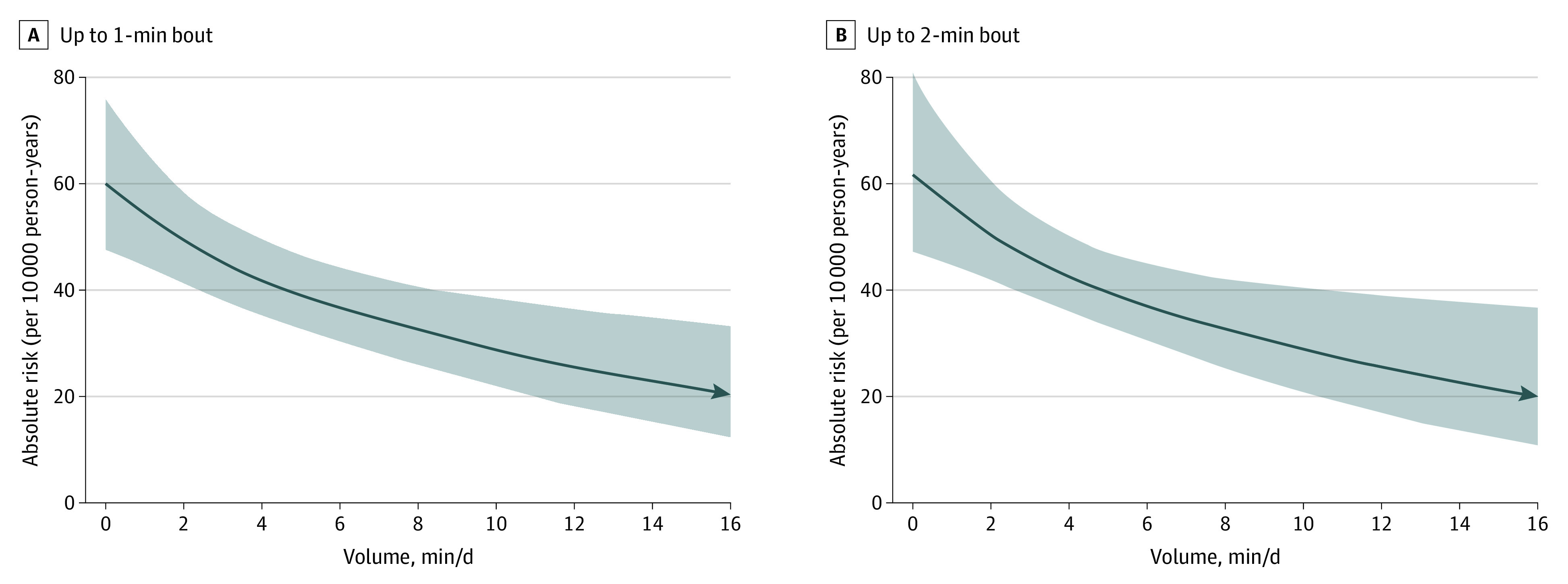
Adjusted Absolute Risk Dose-Response of Vigorous Intermittent Lifestyle Physical Activity (VILPA) Daily Duration, From Bouts of Up to 1 and 2 Minutes, With Physical Activity–Related Cancer Incidence (n = 22 398; 1084 Events) Absolute risk adjusted for age, sex, body mass index (calculated as weight in kilograms divided by height in meters squared), duration of light-intensity physical activity, duration of moderate-intensity physical activity, smoking status, alcohol consumption, accelerometer-estimated sleep duration, fruit and vegetable consumption, education level, medication use, self-reported parental history of cancer, and prevalent cardiovascular disease. All analyses were additionally adjusted for vigorous physical activity duration of more than 1 or 2 minutes, as appropriate.

**Figure 2.**
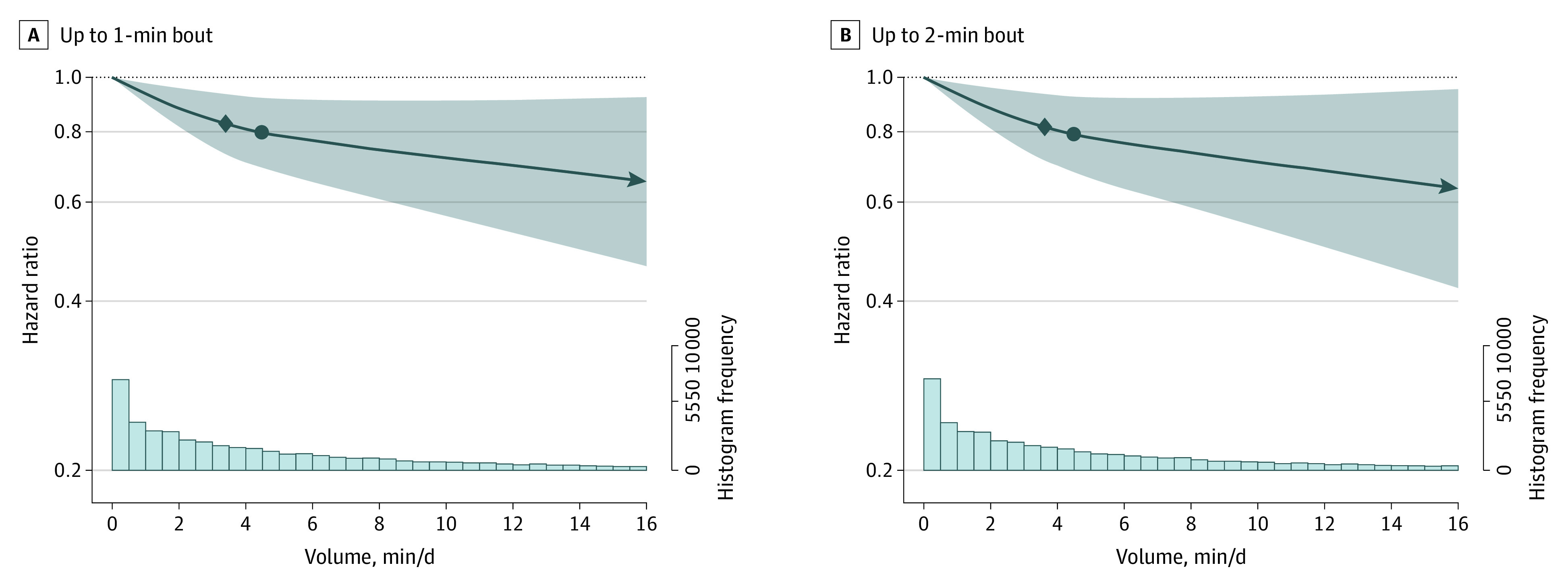
Dose-Response Association of Vigorous Intermittent Lifestyle Physical Activity (VILPA) Daily Duration, From Bouts of Up to 1 and 2 Minutes, With Total Cancer Incidence (n = 22 398; 2356 Events) The diamond shape indicates the ED_50_ value, the minimal dose defined as the daily duration of VILPA associated with 50% of the optimal risk reduction; and the circle, the effect associated with the median VILPA value (the list of values is available in eTable 7 in Supplement 1) . Analyses were adjusted for age, sex, body mass index (calculated as weight in kilograms divided by height in meters squared), duration of light-intensity physical activity, duration of moderate-intensity physical activity, smoking status, alcohol consumption, accelerometer- estimated sleep duration, fruit and vegetable consumption, education level, medication use, self-reported parental history of cancer, and prevalent cardiovascular disease. All analyses were additionally adjusted for vigorous physical activity duration of more than 1 (bouts up to 1 minute exposure) minute or more than 2 (bouts up to 2 minutes exposure) minutes. Hazard ratios were calculated using Fine-Gray models.

**Figure 3.**
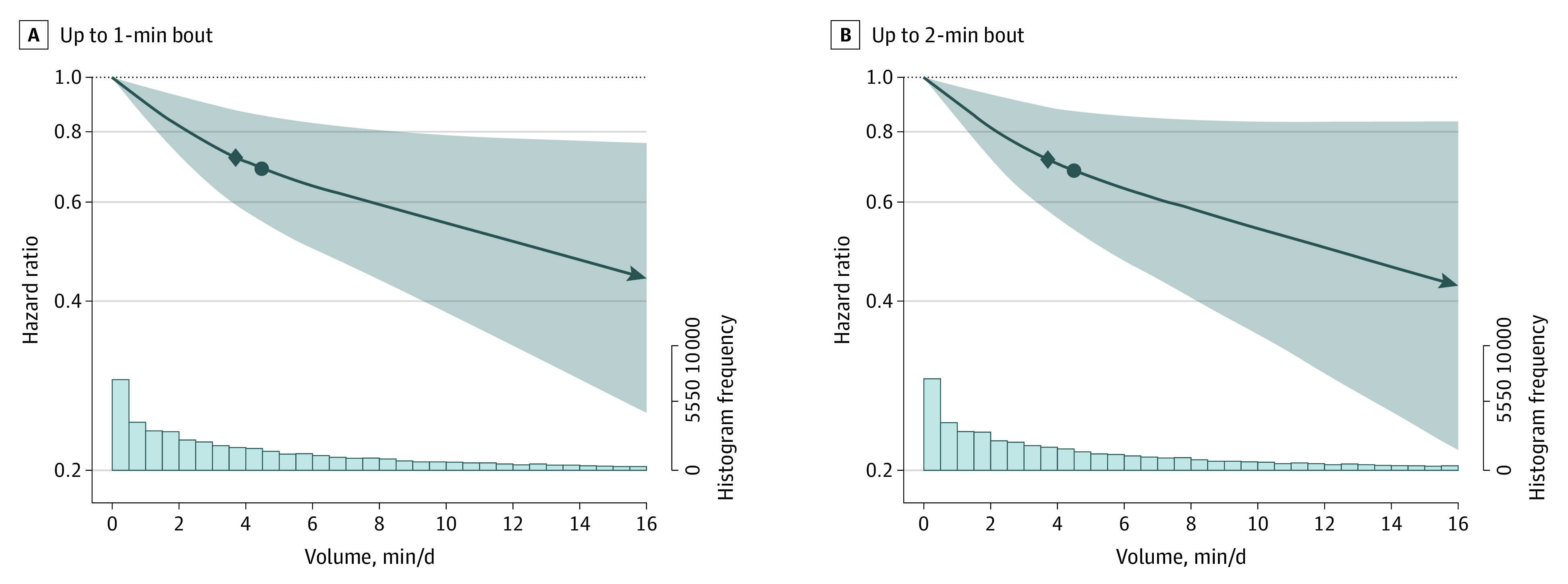
Dose-Response Association of Vigorous Intermittent Lifestyle Physical Activity (VILPA) Daily Duration From Bouts of Up to 1 and 2 Minutes With Physical Activity–Related Cancer Incidence (n = 22 398; 1084 Events) The diamond shape indicates the ED_50_ value, the minimal dose defined as the daily duration of VILPA associated with 50% of the optimal risk reduction; and the circle, the effect associated with the median VILPA value (the list of values is available in eTable 7 in Supplement 1) Analyses were adjusted for age, sex, body mass index (calculated as weight in kilograms divided by height in meters squared), duration of light-intensity physical activity, duration of moderate-intensity physical activity, smoking status, alcohol consumption, accelerometer-estimated sleep duration, fruit and vegetable consumption, education, medication use, self-reported parental history of cancer, and prevalent cardiovascular. All analyses were additionally adjusted for vigorous physical activity duration of more than 1 (bouts up to 1 minute exposure) minute or more than 2 (bouts up to 2 minutes exposure) minutes. Hazard ratios were calculated using Fine-Gray models.

Most (92.3%) VILPA was accrued in bouts of up to 1 min while 97.3% of all bouts lasted up to 2 min. For both duration lengths, the median daily average VILPA was 4.5 min and the maximum was 16.0 min. Adjusted dose-response curves of absolute risk (eFigure 2 in Supplement 1; Figure 1) and relative hazard ratios (HR) indicated a near-linear association of both VILPA bout lengths with total and PA-related cancer incidence (Figures 2 and 3). The dose-response curves were steeper, and the magnitude of the risk reduction sharper for PA-related cancer than for total cancer incidence. For example, the minimum dose^4,6,10^ for VILPA bouts of up to 1 minute was 3.4 (total cancer) min/d and 3.7 (PA-related cancer) min/d (HRs, 0.83; 95% CI, 0.73-0.93; and HR, 0.72; 95% CIs, 0.59-0.88, respectively) (eTable 7 and eFigures 13 and 14 in Supplement 1). The daily median VILPA duration (4.5 min/d) was associated with an HR of 0.80 (95% CI, 0.69-0.92) and 0.69 (95% CI, 0.55-0.86).

Results for VILPA bouts of up to 2 min were very similar. In the sensitivity analyses, excluding participants who were underweight or in poor health or had an event in the first 2 years (eFigure 3 in Supplement 1) and removing BMI from the models (eFigure 4 in Supplement 1) did not appreciably change the results. For the estimates to be attenuated to null, the association of an unmeasured confounder with exposures and incident cancer would need to have a HR of 1.70 (95% CI lower bound, 1.36) and 2.12 (95% CI lower bound, 1.53) for PA-related cancer (eTable 8 in Supplement 1). Additional adjustment for VILPA energy expenditure (kilojoules/kg/d) had minimal effect on the dose-response curves (eFigure 5 in Supplement 1). eFigure 6 in Supplement 1 presents the nested models sequentially adjusted for groups of confounders, and eFigure 7 in Supplement 1 presents a categorical VILPA exposure analysis. Adjustment for smoking pack-years revealed a modest attenuation of the associations for PA-related cancer (eFigures 8 and 9 in Supplement 1). Adjustments for detailed alcohol consumption (eFigures 10 and 11 in Supplement 1) and for prevalent diabetes (eFigures 12 and 13 in Supplement 1) showed minimal associations.

## Discussion

Nonexercising adults, the majority of the middle-aged population,^2,11^ are at an increased risk of developing certain cancers.^7^ We found inverse associations of modest VILPA amounts with total cancer and, in a more pronounced manner, PA-related cancer incidence. Although steeper risk reductions occurred at the lower end of the VILPA distribution (up to approximately 4-5 min/d), there were continuing gains with higher daily VILPA amounts. With little variation between bouts of up to 1 or 2 min, a minimum of 3.4 to 3.6 min of VILPA/d was associated with a 17% to 18% reduction in total incident cancer risk (compared with no VILPA). The study sample median of 4.5 VILPA min/d was associated with a 31% to 32% reduction in PA-related cancer incidence. For comparison, 1 metabolic equivalent unit increase in cardiorespiratory fitness (3.5 mL of oxygen uptake/kg/min) is associated with a 7% reduction in total cancer risk.^12^

Proof-of-concept trials^13^ have shown that small doses of intermittent VPA may produce rapid improvements in cardiorespiratory fitness, providing a potential biological explanation of the associations with incident cancer mortality observed in the present study findings. More VPA has also been specifically associated with reduced risk of breast, endometrial, and colon cancers.^1^ The main biologic pathways associating PA and cancer incidence are inflammation, insulin resistance, body composition, and endogenous sex hormones.^14^ Although the present and previous studies were observational and could not confirm causation, a recent Mendelian randomization analysis^15^ provided evidence of a causal association between VPA and breast cancer. To our knowledge, this study is among the first to use a wrist accelerometry classifier to estimate VILPA.^4,6^

### Limitations

A limitation of this study was that responses to the leisure-time exercise questions, which determined the sample inclusion criteria,^4^ were administered an average of 5.5 years before the accelerometry baseline was recorded. However, the questions had high stability over time (88%) among the 6095 participants who had repeated examinations.^4^

## Conclusions

This cohort study found that daily VILPA duration was inversely associated with incident cancer risk in a near-linear manner, with steeper dose-response for PA-related cancers. As few as 4 to 5 min of VILPA daily was associated with a substantially lower cancer risk. Long-term trials with cancer-related biomarker outcomes and well-designed cohort studies with wearable devices should further explore the potential of VILPA as a cancer prevention intervention for nonexercising individuals and for those who find structured exercise unappealing.

## Notes

Supplement 1. eTable 1. Questions to assess participation in leisure time physical activity eTable 2. Characteristics of excluded non-exercisers eTable 3. Number of events per physical activity-related cancer site eTable 4. Covariate definitions eTable 5. STROBE Statement eTable 6. Sample characteristics by tertiles of average daily duration of up to 1 minute bouts of vigorous intermittent lifestyle physical activity (VILPA) in a sample of UK Biobank participants reporting no exercise or sports (N=22,398). eTable 7. Hazard ratios associated with the minimum dose1 and median and maximum VILPA values eTable 8. E-values for minimum dose, and median/maximum VILPA values for incident cancer eFigure 1. Flow diagram of participants in the study eFigure 2. Adjusted absolute risk dose-response of VILPA with total incident cancer eFigure 3. Dose-response of up to one-minute VILPA duration with exclusion for poor health, underweight, and cancer events occurring during the first 2 years of follow up eFigure 4. Dose-response of up to one-minute VILPA duration with further adjustment for body mass index eFigure 5. Dose-response of up to one-minute VILPA duration with adjustment for VILPA energy expenditure (KJ/kg per day) eFigure 6. Sequential dose-response of up to 1-minute VILPA duration eFigure 7. Categorical analysis of VILPA with total cancer and PA-related cancer eFigure 8. Dose-response association of Vigorous Intermittent Lifestyle Physical Activity (VILPA) daily duration from bouts lasting up to one and two minutes with total cancer incidence; adjustment for smoking pack-years (n=19,103; events=1,962). for direct comparisons with the main analyses we also present results in the same sample of the analyses adjusted for the existing smoking status variable eFigure 9. Dose-response association of Vigorous Intermittent Lifestyle Physical Activity (VILPA) daily duration from bouts lasting up to one and two minutes with PA-related cancer incidence; adjustment for smoking pack-years (n=19,103; events=913). For a direct comparisons with the main analyses we also present results of the analyses adjusted for the existing smoking status variable in the same sample eFigure 10. Dose-response association of Vigorous Intermittent Lifestyle Physical Activity (VILPA) daily duration from bouts lasting up to one and two minutes with total cancer incidence; adjustment for units per day of alcohol (See eTable 4). eFigure 11. Dose-response association of Vigorous Intermittent Lifestyle Physical Activity (VILPA) daily duration from bouts lasting up to one and two minutes with PA-related incidence; adjustment for units per day of alcohol eFigure 12. Dose-response association of Vigorous Intermittent Lifestyle Physical Activity (VILPA) daily duration from bouts lasting up to one and two minutes with total cancer incidence; with adjustment for prevalent diabetes eFigure 13. Dose-response association of Vigorous Intermittent Lifestyle Physical Activity (VILPA) daily duration from bouts lasting up to one and two minutes with PA-related incidence; adjustment for prevalent diabetes eFigure 14. Dose-response association of Vigorous Intermittent Lifestyle Physical Activity (VILPA) daily duration from bouts lasting up to one and two minutes with total cancer incidence; stratified by sex (women n=12,276 ; men n= 10,122 ). eAppendix. Follow up ascertainment descriptives, unabridged sampling, design, and physical activity-related methods

Supplement 2. Data Sharing Statement

## References

[1] Matthews CE, Moore SC, Arem H, . Amount and intensity of leisure-time physical activity and lower cancer risk. J Clin Oncol. 2020;38(7):686-697. doi:10.1200/JCO.19.02407 31877085 PMC7048166

[2] Oja P, Kelly P, Pedisic Z, . Associations of specific types of sports and exercise with all-cause and cardiovascular-disease mortality: a cohort study of 80 306 British adults. Br J Sports Med. 2017;51(10):812-817. doi:10.1136/bjsports-2016-096822 27895075

[3] Stamatakis E, Huang BH, Maher C, . Untapping the health enhancing potential of vigorous intermittent lifestyle physical activity (VILPA): rationale, scoping review, and a 4-pillar research framework. Sports Med. 2021;51(1):1-10. doi:10.1007/s40279-020-01368-8 33108651 PMC7806564

[4] Stamatakis E, Ahmadi MN, Gill JMR, . Association of wearable device-measured vigorous intermittent lifestyle physical activity with mortality. Nat Med. 2022;28(12):2521-2529. doi:10.1038/s41591-022-02100-x 36482104 PMC9800274

[5] Ramakrishnan R, Doherty A, Smith-Byrne K, . Accelerometer measured physical activity and the incidence of cardiovascular disease: Evidence from the UK Biobank cohort study. [published correction appears in PLoS Med. 2021;18(9):e1003809]. PLoS Med. 2021;18(1):e1003487. doi:10.1371/journal.pmed.1003487 33434193 PMC7802951

[6] Ahmadi MN, Clare PJ, Katzmarzyk PT, Del Pozo Cruz B, Lee IM, Stamatakis E. Vigorous physical activity, incident heart disease, and cancer: how little is enough? Eur Heart J. 2022;43(46):4801-4814. doi:10.1093/eurheartj/ehac572 36302460 PMC9726449

[7] Moore SC, Lee IM, Weiderpass E, . Association of Leisure-Time Physical Activity With Risk of 26 Types of Cancer in 1.44 Million Adults. JAMA Intern Med. 2016;176(6):816-825. doi:10.1001/jamainternmed.2016.1548 27183032 PMC5812009

[8] Austin PC, Fine JP. Practical recommendations for reporting Fine-Gray model analyses for competing risk data. Stat Med. 2017;36(27):4391-4400. doi:10.1002/sim.7501 28913837 PMC5698744

[9] Ahmadi MN, Nathan N, Sutherland R, Wolfenden L, Trost SG. Non-wear or sleep? evaluation of five non-wear detection algorithms for raw accelerometer data. J Sports Sci. 2020;38(4):399-404. doi:10.1080/02640414.2019.1703301 31826746

[10] Rampinelli C, De Marco P, Origgi D, . Exposure to low dose computed tomography for lung cancer screening and risk of cancer: secondary analysis of trial data and risk-benefit analysis. BMJ. 2017;356:j347. doi:10.1136/bmj.j347 28179230 PMC5421449

[11] Bennie JA, Pedisic Z, van Uffelen JG, . The descriptive epidemiology of total physical activity, muscle-strengthening exercises and sedentary behaviour among Australian adults--results from the National Nutrition and Physical Activity Survey. BMC Public Health. 2016;16:73. doi:10.1186/s12889-016-2736-3 26809451 PMC4727339

[12] Han M, Qie R, Shi X, . Cardiorespiratory fitness and mortality from all causes, cardiovascular disease and cancer: dose-response meta-analysis of cohort studies. Br J Sports Med. 2022;56(13):733-739. doi:10.1136/bjsports-2021-104876 35022163

[13] Allison MK, Baglole JH, Martin BJ, Macinnis MJ, Gurd BJ, Gibala MJ. Brief intense stair climbing improves cardiorespiratory fitness. Med Sci Sports Exerc. 2017;49(2):298-307. doi:10.1249/MSS.0000000000001188 28009784

[14] Friedenreich CM, Ryder-Burbidge C, McNeil J. Physical activity, obesity and sedentary behavior in cancer etiology: epidemiologic evidence and biologic mechanisms. Mol Oncol. 2021;15(3):790-800. doi:10.1002/1878-0261.12772 32741068 PMC7931121

[15] Dixon-Suen SC, Lewis SJ, Martin RM, ; Breast Cancer Association Consortium. Physical activity, sedentary time and breast cancer risk: a Mendelian randomisation study. Br J Sports Med. 2022;56(20):1157-1170. doi:10.1136/bjsports-2021-105132 36328784 PMC9876601

