# Supplementary material for: Vigorous Intermittent Lifestyle Physical Activity and Cancer Incidence Among Nonexercising Adults: The UK Biobank Accelerometry Study
Source: JAMA Oncol. 2023 Jul 27;9(9):1255–9. doi: 10.1001/jamaoncol.2023.1830 (PMC10375384; doi:10.1001/jamaoncol.2023.1830)
Supplement: Supplement 1. — eTable 1. Questions to assess participation in leisure time physical activity eTable 2. Characteristics of excluded non-exercisers eTable 3. Number of events per physical activity-related cancer site eTable 4. Covariate definitions eTable 5. STROBE Statement eTable 6. Sample characteristics by tertiles of average daily duration of up to 1 minute bouts of vigorous intermittent lifestyle physical activity (VILPA) in a sample of UK Biobank participants reporting no exercise or sports (N=22,398). eTable 7. Hazard ratios associated with the minimum dose1 and median and maximum VILPA values eTable 8. E-values for minimum dose, and median/maximum VILPA values for incident cancer eFigure 1. Flow diagram of participants in the study eFigure 2. Adjusted absolute risk dose-response of VILPA with total incident cancer eFigure 3. Dose-response of up to one-minute VILPA duration with exclusion for poor health, underweight, and cancer events occurring during the first 2 years of follow up eFigure 4. Dose-response of up to one-minute VILPA duration with further adjustment for body mass index eFigure 5. Dose-response of up to one-minute VILPA duration with adjustment for VILPA energy expenditure (KJ/kg per day) eFigure 6. Sequential dose-response of up to 1-minute VILPA duration eFigure 7. Categorical analysis of VILPA with total cancer and PA-related cancer eFigure 8. Dose-response association of Vigorous Intermittent Lifestyle Physical Activity (VILPA) daily duration from bouts lasting up to one and two minutes with total cancer incidence; adjustment for smoking pack-years (n=19,103; events=1,962). for direct comparisons with the main analyses we also present results in the same sample of the analyses adjusted for the existing smoking status variable eFigure 9. Dose-response association of Vigorous Intermittent Lifestyle Physical Activity (VILPA) daily duration from bouts lasting up to one and two minutes with PA-related cancer incidence; adjustment for smoking pack-years (n=19,10 [file jamaoncol-e231830-s001.pdf]

Supplemental Online Content

Stamatakis E, Ahmadi MN, Friedenreich CM, et al. Vigorous intermittent lifestyle physical activity and cancer incidence among nonexercising adults: the UK Biobank accelerometry study. *JAMA Oncol*. Published July 27, 2023. doi:10.1001/jamaoncol.2023.1830

eTable 1. Questions to assess participation in leisure time physical activity

eTable 2. Characteristics of excluded non-exercisers

eTable 3. Number of events per physical activity-related cancer site

eTable 4. Covariate definitions

eTable 5. STROBE Statement

eTable 6. Sample characteristics by tertiles of average daily duration of up to 1 minute bouts of vigorous intermittent lifestyle physical activity (VILPA) in a sample of UK Biobank participants reporting no exercise or sports (N=22,398)

eTable 7. Hazard ratios associated with the minimum dose1 and median and maximum VILPA values

eTable 8. E-values for minimum dose, and median/maximum VILPA values for incident cancer

eFigure 1. Flow diagram of participants in the study

eFigure 2. Adjusted absolute risk dose-response of VILPA with total incident cancer

eFigure 3. Dose-response of up to one-minute VILPA duration with exclusion for poor health, underweight, and cancer events occurring during the first 2 years of follow up

eFigure 4. Dose-response of up to one-minute VILPA duration with further adjustment for body mass index

eFigure 5. Dose-response of up to one-minute VILPA duration with adjustment for VILPA energy expenditure (KJ/kg per day)

eFigure 6. Sequential dose-response of up to 1-minute VILPA duration

eFigure 7. Categorical analysis of VILPA with total cancer and PA-related cancer

eFigure 8. Dose-response association of Vigorous Intermittent Lifestyle Physical Activity (VILPA) daily duration from bouts lasting up to one and two minutes with total cancer incidence; adjustment for smoking pack-years (n=19,103; events=1,962). For direct comparisons with the main analyses we also present results in the same sample of the analyses adjusted for the existing smoking status variable

eFigure 9. Dose-response association of Vigorous Intermittent Lifestyle Physical Activity (VILPA) daily duration from bouts lasting up to one and two minutes with PA-related cancer incidence; adjustment for smoking pack-years (n=19,103; events=913). For a direct comparisons with the main analyses we also present results of the analyses adjusted for the existing smoking status variable in the same sample

© 2023 Stamatakis E et al. *JAMA Oncol*

eFigure 10. Dose-response association of Vigorous Intermittent Lifestyle Physical Activity (VILPA) daily duration from bouts lasting up to one and two minutes with total cancer incidence; adjustment for units per day of alcohol (See eTable 4)

eFigure 11. Dose-response association of Vigorous Intermittent Lifestyle Physical Activity (VILPA) daily duration from bouts lasting up to one and two minutes with PA-related incidence; adjustment for units per day of alcohol

eFigure 12. Dose-response association of Vigorous Intermittent Lifestyle Physical Activity (VILPA) daily duration from bouts lasting up to one and two minutes with total cancer incidence; with adjustment for prevalent diabetes

eFigure 13. Dose-response association of Vigorous Intermittent Lifestyle Physical Activity (VILPA) daily duration from bouts lasting up to one and two minutes with PA-related incidence; adjustment for prevalent diabetes

eFigure 14. Dose-response association of Vigorous Intermittent Lifestyle Physical Activity (VILPA) daily duration from bouts lasting up to one and two minutes with total cancer incidence; stratified by sex (women n=12,276 ; men n= 10,122)

eAppendix. Follow up ascertainment descriptives, unabridged sampling, design, and physical activity-related methods

This supplemental material has been provided by the authors to give readers additional information about their work.

© 2023 Stamatakis E et al. *JAMA Oncol*

eTable 1: Questions to assess participation in leisure time physical activity

Types of physical activity in last 4 weeks UK Biobank Field ID: 6164:	• Walking for pleasure (not as a means of transport)<br>• Other exercises (eg: swimming, cycling, keep fit, bowling)<br>• Strenuous sports<br>• Light DIY (eg: pruning, watering the lawn)<br>• Heavy DIY (eg: weeding, lawn mowing, carpentry, digging)<br>• None of the above<br>• Prefer not to answer<br>•
Each time you did strenuous sports, about how long did you spend doing it? (UK Biobank field ID: 1001)	• Less than 15 minutes<br>• between 15 and 30 minutes<br>• between 30 minutes and 1 hour<br>• between 1 hour and 1.5 hours<br>• between 1.5 hours and 2 hours between 2 and 3 hours<br>• over 3 hours, do not know<br>• prefer not to answer
How many times in the last 4 weeks did you do strenuous sports? (UK Biobank field ID: 991)	• Once in the last 4 weeks<br>• 2-3 times in the last 4 weeks<br>• Once a week<br>• 2-3 times a week<br>• 4-5 times a week<br>• Every day<br>• Do not know<br>• Prefer not to answer
Each time you did other exercises such as swimming, cycling, keep fit, about how long did you spend doing it? (UK Biobank field ID: 3647)	• Less than 15 minutes<br>• between 15 and 30 minutes<br>• between 30 minutes and 1 hour<br>• between 1 hour and 1.5 hours<br>• between 1.5 hours and 2 hours between 2 and 3 hours<br>• over 3 hours, do not know<br>• prefer not to answer
How many times in the last 4 weeks did you do other exercises such as swimming, cycling, keep fit? (UK Biobank field ID: 3637)	• Once in the last 4 weeks<br>• 2-3 times in the last 4 weeks<br>• Once a week<br>• 2-3 times a week<br>• 4-5 times a week<br>• Every day<br>• Do not know<br>• Prefer not to answer
How many times in the last 4 weeks did you go walking for pleasure? (UK Biobank field ID: 971)	• Once in the last 4 weeks<br>• 2-3 times in the last 4 weeks<br>• Once a week<br>• 2-3 times a week<br>• 4-5 times a week<br>• Every day<br>• Do not know<br>• Prefer not to answer

© 2023 Stamatakis E et al. *JAMA Oncol*

eTable 2: characteristics of the non-exercisers who were excluded from the analyses

	**Included** (n=22,398)	**Excluded*** (n=68,782)
**Follow up in yrs, mean (sd)**	6.7 (1.2)	6.8 (1.2)
**Age, mean (sd)**	62.0 (7.6)	61.9 (7.9)
**Male, n (%)**	10,122 (45.2)	29,754 (43.3)
**Ethnicity, n (%)**		
Asian	303 (1.4)	740 (1.1)
Black	237 (1.1)	519 (0.8)
Mixed	142 (0.6)	354 (0.5)
Other	207 (0.9)	552 (0.8)
White	21,509 (96.0)	66,575 (96.9)
**Smoking history, n (%)**		
Current	2,066 (9.2)	4,177 (6.1)
Never	12,499 (55.8)	39,492 (57.6)
Previous	7,833 (35.0)	24,893 (36.3)
[a]Smoking pack years, n (%)		
Current and <22 pack years	474 (2.1)	1,004 (1.5)
Current and 22 to 38 pack years	494 (2.2)	723 (1.1)
Current and >38 pack years	502 (2.2)	606 (0.9)
Never	12,499 (55.8)	39,545 (57.7)
Previous and <11 pack years	1,656 (7.4)	5,977 (8.7)
Previous and 11 to 25 pack years	1,854 (8.3)	5,878 (8.6)
Previous and >25 pack years	1,687 (7.5)	3,878 (5.7)
**Alcohol consumption, n (%)**		
Never	861 (3.8)	1,781 (2.6)
Ex-drinker	752 (3.4)	1,728 (2.5)
Within guidelines	13,278 (59.3)	38,406 (56.2)
Above guidelines	7,507 (33.5)	26,376 (38.6)
**Education, n (%)**		
College/University	8,081 (36.1)	31,189 (45.3)
A/AS level	2,854 (12.7)	9,048 (13.2)
O level	4,904 (21.9)	13,607 (19.8)
CSE	1,178 (5.3)	2,419 (3.5)
NVQ/HND/HNC	1,420 (6.3)	3,464 (5.0)
Other	3,961 (17.7)	9,055 (13.2)
**Fruit and vegetable consumption**	7.4 (4.2)	8.4 (4.5)
**Prevalent CVD, n (%)**	2,342 (10.5)	6,414 (9.3)
**Family history of cancer, n (%)**	5,596 (25.0)	17,364 (25.2)
**Medication, n (%)**		
Cholesterol	3,781 (16.9)	9,407 (13.7)

Blood pressure	4,438 (19.8)	10,999 (16.0)
Insulin	205 (0.9)	422 (0.6)
**Sedentary behaviour in mins, median [IQR]**	652 [593, 712]	638 [580, 695]
**Light intensity in mins, median [IQR]**	88.5 [59.5, 136.1]	90.9 [62.7, 139.2]
**Moderate intensity in mins, median [IQR]**	23.8 [13.5, 39.6]	27.8 [16.2, 44.9]
**Vigorous intensity in mins, median [IQR]**	4.1 [1.4, 10.4]	4.8 [2.3, 12.1]
**Sleep duration in mins, median [IQR]**	437 [396, 475]	439 [400, 474]
**VILPA bout duration up to 1 minute, median [IQR]**	3.9 [1.2, 9.1]	4.6 [1.5, 8.5]
**VILPA bout duration up to 2 minute, median [IQR]**	4.1 [1.4, 10.0]	4.8 [2.3, 11.6]

*Due to missing data, values may not add up to full sample; sd – standard deviation; IQR – interquartile range

eTable 3: Number of events per physical activity-related cancer site. Total Cancer Incidence was defined as hospital episode, cancer registration, or death attributed to any cancer excluded in situ, benign, uncertain, non-melanoma skin cancers, or non-well-defined cancers. (ICD-10 codes beginning with 'C0' 'C1' 'C2' 'C3' 'C4' (excluding C49.9) 'C5' 'C6' 'C70' 'C71' 'C72' 'C73' 'C74' 'C75' 'C7A' 'C8' 'C9')).

Physical activity-related cancer incidence included the following 13 sites:	n
Esophageal adenocarcinoma	59
Liver	47
Lung	114
Kidney	37
Gastric Cardia	29
Endometrial	52
Myeloid Leukemia	39
Myeloma	48
Colorectal*	188
Head and neck	137
Bladder	80
Breast	254
**TOTAL**	**1084**

*Colon and rectal cancer have been collapsed into colorectal in accordance with UKBB linkage (https://biobank.ndph.ox.ac.uk/~bbdatan/DeathSummaryReport.html)

© 2023 Stamatakis E et al. *JAMA Oncol*

eTable 4: Covariate definitions

Variable	Definition	UK Biobank field ID (if applicable)
Age	Continuous	34, 52, accelerometer date-timestamp
Sex	Female/Male	31
Light intensity	Continuous<br>Ambulatory activities <3 METs	Derived from accelerometer data (see Supplemental Text methods)
Moderate intensity	Continuous<br>Brisk walking, energetic activities (≥3 to <6 METs)	Derived from accelerometer data (see Supplemental Text methods)
VILPA Duration from longer bouts	VILPA Duration from bouts lasting over 1 or 2 minutes<br>Eg, for the analysis of bouts lasting up to 2 minutes in duration, this variable contained VILPA duration from bouts that lasted more than 2 minutes in duration	Derived from accelerometer data (see Supplemental Text methods)
Smoking status	Never, past, current	20116
Alcohol consumption (main analyses\)	Never, ex-drinker, within guidelines drinker (up to 14 units a week), above guidelines drinker (>14 units a week). 1 unit = 8 grams of ethanol	20117, 1558
Alcohol consumption (sensitivity analyses)	Never drinker, Ex-drinker<br>Within guidelines and <1 unit/wk, Within guidelines and 1 to 4 units/wk, Within guidelines and >4 units/wk, Above guidelines and <15 units/wk, Above guidelines and ≥15 units/wk	
Sleep duration	Hours spent sleeping	Derived from accelerometer data (see Online Methods)
Diet	Fruits and vegetables servings/day	1309, 1319, 1289, 1299
*Prevalent cancer	Identified by self-report and cancer registry	20001, 100092
Prevalent CVD	Yes/No<br>Identified by self-report and hospitalisation. The ICD-10 codes included were: I0, I11, I13, I20-I51, I60-I69.	20002, 41259
Education	College/University; A/AS level; O levels; CSE;<br>NVQ/HND/HNC; other.	6138

© 2023 Stamatakis E et al. *JAMA Oncol*

	A/AS = Advanced Placement;<br>O level = High school certificate;<br>CSE = Certificate of secondary education;<br>NVQ/HND/HNC = Vocational qualification / Associates degree.<br><br>A level, typically at age 18 years;<br>O level, typically at age 16 years;<br>CSE, typically at age 16 years;<br><br>These categories correspond roughly to the US education levels: :<br>A/AS = Advanced Placement<br>O level = High school certificate<br>CSE = Certificate of secondary education<br>NVQ/HND/HNC = Vocational qualification / Associates degree	
Use of cholesterol medication	Yes/No	6177, 6153
Use of blood pressure medication	Yes/No	6177, 6153
Use of diabetes medication	Yes/No	6177, 6153
Parental history of cancer	Yes/No<br>Self-reported mother or father diagnosed with cancer	20107, 20110
Adiposity	Body mass index (body weight/height$^{2)}$; continuous	21001
Prevalent diabetes	Yes/No<br>Identified by general practitioner and hospitalisation records. The ICD 10 code used was E11	42040, 41259

*Used to exclude participants

© 2023 Stamatakis E et al. *JAMA Oncol*

eTable 5: STROBE Statement

	Item No	Recommendation	Page No
**Title and abstract**	1	(*a*) Indicate the study's design with a commonly used term in the title or the abstract	1, 4
		(*b*) Provide in the abstract an informative and balanced summary of what was done and what was found	4
**Introduction**			
Background/rationale	2	Explain the scientific background and rationale for the investigation being reported	5
Objectives	3	State specific objectives, including any prespecified hypotheses	5
**Methods**			
Study design	4	Present key elements of study design early in the paper	5
Setting	5	Describe the setting, locations, and relevant dates, including periods of recruitment, exposure, follow-up, and data collection	5
Participants	6	(*a*) *Cohort study*—Give the eligibility criteria, and the sources and methods of selection of participants. Describe methods of follow-up<br>*Case-control study*—Give the eligibility criteria, and the sources and methods of case ascertainment and control selection. Give the rationale for the choice of cases and controls<br>*Cross-sectional study*—Give the eligibility criteria, and the sources and methods of selection of participants	5
		(*b*) *Cohort study*—For matched studies, give matching criteria and number of exposed and unexposed<br>*Case-control study*—For matched studies, give matching criteria and the number of controls per case	NA
Variables	7	Clearly define all outcomes, exposures, predictors, potential confounders, and effect modifiers. Give diagnostic criteria, if applicable	5
Data sources/ measurement	8*	For each variable of interest, give sources of data and details of methods of assessment (measurement). Describe comparability of assessment methods if there is more than one group	5

Bias	9	Describe any efforts to address potential sources of bias	5, 6
Study size	10	Explain how the study size was arrived at	5, eFigure 1
Quantitative variables	11	Explain how quantitative variables were handled in the analyses. If applicable, describe which groupings were chosen and why	5, 6
Statistical methods	12	(*a*) Describe all statistical methods, including those used to control for confounding	5, 6
		(*b*) Describe any methods used to examine subgroups and interactions	NA
		(*c*) Explain how missing data were addressed	5
		(*d*) *Cohort study*—If applicable, explain how loss to follow-up was addressed<br>*Case-control study*—If applicable, explain how matching of cases and controls was addressed<br>*Cross-sectional study*—If applicable, describe analytical methods taking account of sampling strategy	NA
		(*e*) Describe any sensitivity analyses	6
**Results**			
Participants	13*	(a) Report numbers of individuals at each stage of study—eg numbers potentially eligible, examined for eligibility, confirmed eligible, included in the study, completing follow-up, and analysed	eFigure 1
		(b) Give reasons for non-participation at each stage	eFigure 1
		(c) Consider use of a flow diagram	eFigure 1
Descriptive data	14*	(a) Give characteristics of study participants (eg demographic, clinical, social) and information on exposures and potential confounders	eTable 6
		(b) Indicate number of participants with missing data for each variable of interest	eFigure 1
		(c) *Cohort study*—Summarise follow-up time (eg, average and total amount)	6
Outcome data	15*	*Cohort study*—Report numbers of outcome events or summary measures over time	6
		Case-control study—Report numbers in each exposure category, or summary measures of exposure	*NA*

© 2023 Stamatakis E et al. *JAMA Oncol*

		Cross-sectional study—Report numbers of outcome events or summary measures	*NA*
Main results	16	(*a*) Give unadjusted estimates and, if applicable, confounder-adjusted estimates and their precision (eg, 95% confidence interval). Make clear which confounders were adjusted for and why they were included	6
		(*b*) Report category boundaries when continuous variables were categorized	NA
		(*c*) If relevant, consider translating estimates of relative risk into absolute risk for a meaningful time period	NA
Other analyses	17	Report other analyses done—eg analyses of subgroups and interactions, and sensitivity analyses	6
**Discussion**			
Key results	18	Summarise key results with reference to study objectives	7
Limitations	19	Discuss limitations of the study, taking into account sources of potential bias or imprecision. Discuss both direction and magnitude of any potential bias	7
Interpretation	20	Give a cautious overall interpretation of results considering objectives, limitations, multiplicity of analyses, results from similar studies, and other relevant evidence	7
Generalisability	21	Discuss the generalisability (external validity) of the study results	7
**Other information**			
Funding	22	Give the source of funding and the role of the funders for the present study and, if applicable, for the original study on which the present article is based	9

eTable 6: Sample characteristics by rounded tertiles of average daily duration of up to 1 minute bouts of vigorous intermittent lifestyle physical activity (VILPA) in a sample of UK Biobank participants reporting no exercise or sports (N=22,398), and comparisons with full sample with valid data (including exercisers).

	**No VILPA** n=1,392 (6.2%)	**>0 to <3 (mins/d)** n=8,168 (36.5%)	**>3 to <8 (mins/d)** n=6,346 (28.3%)	**>=8(mins/d)** n=6,492 (29.0%)	**Overall (VILPA Sample)** n=22,398	**Overall* eligible sample** (**(n=76,956**)
**Follow up in yrs, mean (SD)**	6.5 (1.4)	6.7 (1.3)	6.7 (1.2)	6.7 (1.2)	6.7 (1.2)	6.8 (1.0)
**Age, mean (SD)**	65.2 (6.8)	63.2 (7.4)	61.5 (7.6)	60.2 (7.6)	62.0 (7.6)	61.4 (7.9)
**Male, n (%)**	449 (32.3)	3,082 (37.7)	2,953 (46.5)	3,638 (56.0)	10,122 (45.2)	34,362 (44.7)
**Ethnicity, n (%)**						
Asian	10 (0.7)	100 (1.2)	90 (1.4)	103 (1.6)	303 (1.4)	895 (1.2)
Black	10 (0.7)	73 (0.9)	63 (1.0)	91 (1.4)	237 (1.1)	653 (0.8)
Mixed	8 (0.6)	46 (0.6)	38 (0.6)	50 (0.8)	142 (0.6)	423 (0.5)
Other	11 (0.8)	79 (1.0)	44 (0.7)	73 (1.1)	207 (0.9)	632 (0.8)
White	1,353 (97.2)	7,870 (96.4)	6,111 (96.3)	6,175 (95.1)	21,509 (96.0)	74,353 (96.6)
**Smoking history, n (%)**						
Current	180 (12.9)	796 (9.7)	540 (8.5)	550 (8.5)	2,066 (9.2)	5,276 (6.9)
Never	694 (49.9)	4,462 (54.6)	3,603 (56.8)	3,740 (57.6)	12,499 (55.8)	44,484 (57.8)
Previous	518 (37.2)	2,910 (35.6)	2,203 (34.7)	2,202 (33.9)	7,833 (35.0)	27,196 (35.3)
[a]Smoking pack years, n (%)						
Current and <22 pack years	31 (2.2)	178 (2.2)	128 (2.0)	137 (2.1)	474 (2.1)	1,276 (1.7)
Current and 22 to 38 pack years	54 (3.9)	222 (2.7)	113 (1.8)	105 (1.6)	494 (2.2)	900 (1.2)
Current and >38 pack years	53 (3.8)	200 (2.4)	111 (1.7)	138 (2.1)	502 (2.2)	1,038 (1.3)
Never	694 (49.9)	4,462 (54.6)	3,603 (56.8)	3,740 (57.6)	12,499 (55.8)	44,484 (57.8)

© 2023 Stamatakis E et al. *JAMA Oncol*

Previous and <11 pack years	94 (6.8)	606 (7.4)	467 (7.4)	489 (7.5)	1,656 (7.4)	6,398 (8.3)
Previous and 11 to 25 pack years	109 (7.8)	667 (8.2)	533 (8.4)	545 (8.4)	1,854 (8.3)	6,446 (8.4)
Previous and >25 pack years	161 (11.6)	683 (8.4)	454 (7.2)	389 (6.0)	1,687 (7.5)	4,511 (5.9)
**Alcohol consumption, n (%)**						
Never	62 (4.5)	348 (4.3)	223 (3.5)	228 (3.5)	861 (3.8)	2,206 (2.9)
Ex-drinker	61 (4.4)	322 (3.9)	192 (3.0)	177 (2.7)	752 (3.4)	2,071 (2.7)
Within guidelines	877 (63.0)	4,980 (61.0)	3,718 (58.6)	3,703 (57.0)	13,278 (59.3)	43,770 (56.9)
Above guidelines	392 (28.2)	2,518 (30.8)	2,213 (34.9)	2,384 (36.7)	7,507 (33.5)	28,909 (37.6)
**Education, n (%)**						
College/University	487 (35.0)	2,935 (35.9)	2,299 (36.2)	2,360 (36.4)	8,081 (36.1)	33,569 (43.6)
A/AS level	185 (13.3)	1,053 (12.9)	797 (12.6)	819 (12.6)	2,854 (12.7)	10,186 (13.2)
O level	313 (22.5)	1,757 (21.5)	1,432 (22.6)	1,402 (21.6)	4,904 (21.9)	15,528 (20.2)
CSE	49 (3.5)	393 (4.8)	307 (4.8)	429 (6.6)	1,178 (5.3)	3,118 (4.1)
NVQ/HND/HNC	77 (5.5)	471 (5.8)	408 (6.4)	464 (7.1)	1,420 (6.3)	4,117 (5.3)
Other	281 (20.2)	1,559 (19.1)	1,103 (17.4)	1,018 (15.7)	3,961 (17.7)	10,438 (13.6)
**Fruit and vegetable consumption (servings/day)*, mean (SD)**	7.5 (4.3)	7.4 (4.4)	7.3 (4.0)	7.3 (4.2)	7.4 (4.2)	8.1 (4.4)
**Prevalent CVD, n (%)**	248 (17.8)	985 (12.1)	637 (10.0)	472 (7.3)	2,342 (10.5)	7,006 (9.1)
**Family history of cancer, n (%)**	348 (25.0)	2,071 (25.4)	1,585 (25.0)	1,592 (24.5)	5,596 (25.0)	19,066 (24.8)
**Medication, n (%)**						
Cholesterol	382 (27.4)	1,640 (20.1)	972 (15.3)	787 (12.1)	3,781 (16.9)	10,693 (13.9)
Blood pressure	449 (32.3)	1,921 (23.5)	1,178 (18.6)	890 (13.7)	4,438 (19.8)	12,462 (16.2)
Insulin	32 (2.3)	88 (1.1)	55 (0.9)	30 (0.5)	205 (0.9)	513 (0.7)
Body mass index, kg/m^2	29.8 (6.0)	28.5 (5.4)	27.3 (4.8)	26.4 (4.2)	27.6 (5.1)	26.7 (4.5)
Waist circumference, cm						
Male	104.3 (13.2)	100.8 (12.1)	98.1 (11.2)	95.2 (10.5)	98.2 (11.6)	95.4 (10.8)

© 2023 Stamatakis E et al. *JAMA Oncol*

Female	91.4 (14.1)	87.8 (13.3)	83.9 (12.2)	80.7 (11.0)	85.4 (13.0)	82.5 (11.8)
Body fat percentage	36.5 (8.5)	34.2 (8.5)	31.4 (8.2)	28.5 (7.9)	31.9 (8.6)	30.4 (8.4)
**Sedentary behaviour in mins, median [IQR]**	697 [638, 762]	670 [612, 727]	649 [591, 706]	624 [566, 681]	652.0 [593, 712]	640.0 [581.6, 698.0]
**Light intensity in mins, median [IQR]**	78.9 [51.8, 132.6]	88.0 [57.2, 138.6]	87.2 [59.3, 134.0]	91.5 [63.8, 135.1]	88.5 [59.5, 136.1]	91.5 [62.7, 139.8]
**Moderate intensity in mins, median [IQR]**	10.1 [5.0, 18.8]	17.6 [10.2, 30.0]	25.3 [15.6, 39.1]	35.0 [22.8, 52.8]	23.8 [13.5, 39.6]	27.0 [15.7, 43.6]
**Vigorous intensity in mins, median [IQR]**	-	1.2 [0.6, 2.1]	5.2 [4.0, 6.8]	16.2 [11.5, 26.2]	4.1 [1.4, 10.4]	4.6 [2.0, 11.7]
**Sleep duration, in mins, median [IQR]**	432 [385, 476]	438 [395, 477]	438 [397, 474]	436 [397, 473]	437 [396, 475]	438.0 [399.5, 473.5]
**VILPA bout duration up to 1 minute, median [IQR]**	-	1.2 [0.6, 2.0]	4.9 [3.8, 6.2]	13.9 [10.4, 16.0]	3.9 [1.2, 9.1]	-
**VILPA bout duration up to 2 minute, median [IQR]**	-	1.2 [0.6, 2.0]	5.1 [4.0, 6.7]	15.7 [11.2, 16.0]	4.1 [1.4, 10.0]	-

sd – standard deviation; IQR – interquartile range; *includes fresh and dried fruit, and raw and cooked vegetables. *Sample with valid data (including exercisers); [a]Smoking pack years shown for those with available data

© 2023 Stamatakis E et al. *JAMA Oncol*

eTable 7: Hazard ratios associated with the minimum dose[1] and median VILPA values

A. Total incident cancer

Duration	Dose	HR (95%CI)
up to 1 min bout minimum dose	3.4	0.83 (0.73, 0.93)
up to 1 min bout median VILPA dose	4.5	0.80 (0.69, 0.92)
up to 2 min bout minimum dose	3.6	0.82 (0.71, 0.93)
up to 2 min bout median VILPA dose	4.5	0.79 (0.68, 0.93)

Minimal dose (ED50 value): defined as the duration/frequency of VILPA associated with 50% of the optimal risk reduction. The VILPA duration median values were calculated in the sample excluding participants with zero VILPA. Analyses adjusted for age, sex, duration of light intensity physical activity, duration of moderate intensity physical activity, smoking history, alcohol consumption, accelerometer estimated sleep duration, fruit and vegetable consumption, education, self-reported parental history cancer, and prevalent CVD. All analyses were additionally adjusted for vigorous physical activity duration lasting more than one/two minute as appropriate.

B. Incidence of physical activity-related cancer

Duration	Dose	HR (95%CI)
up to 1 min bout minimum dose	3.7	0.72 (0.59, 0.88)
up to 1 min bout VILPA median	4.5	0.69 (0.55, 0.86)
up to 2 min bout minimum dose	3.7	0.71 (0.58, 0.88)
up to 2 min bout VILPA median	4.5	0.68 (0.53, 0.87)

Minimal dose (ED50 value): defined as the duration/frequency of VILPA associated with 50% of the optimal risk reduction. The VILPA duration median values were calculated in the sample excluding participants with zero VILPA. Analyses adjusted for age, sex, duration of light intensity physical activity, duration of moderate intensity physical activity, smoking history, alcohol consumption, accelerometer estimated sleep duration, fruit and vegetable consumption, education, self-reported parental history cancer, and prevalent CVD. All analyses were additionally adjusted for vigorous physical activity duration/bouts lasting more than one/two minute as appropriate.

© 2023 Stamatakis E et al. *JAMA Oncol*

eTable 8: E-values for minimum dose, and median/maximum VILPA values for incident cancer

A. Total cancer incidence

	**E-Value**
**Duration**	
up to 1 min bout minimum dose	1.70 (1.36)
up to 1 min bout VILPA median	1.81 (1.39)
up to 2 min bout minimum dose	1.74 (1.36)
up to 2 min bout VILPA median	1.85 (1.36)

B. Physical activity-related cancer incidence

	**E-Value**
**Duration**	
up to 1 min bout minimum dose	2.12 (1.53)
up to 1 min bout VILPA median	2.26 (1.60)
up to 2 min bout minimum dose	2.17 (1.53)
up to 2 min bout VILPA median	2.30 (1.56)

Values represent: point estimate (and lower limit of the confidence interval in brackets) that an unmeasured confounder would need to have with both the exposure and outcome, conditional on the measured covariates to explain away the exposure-outcome association

© 2023 Stamatakis E et al. *JAMA Oncol*

eFigure 1: Flow diagram of participants in the study

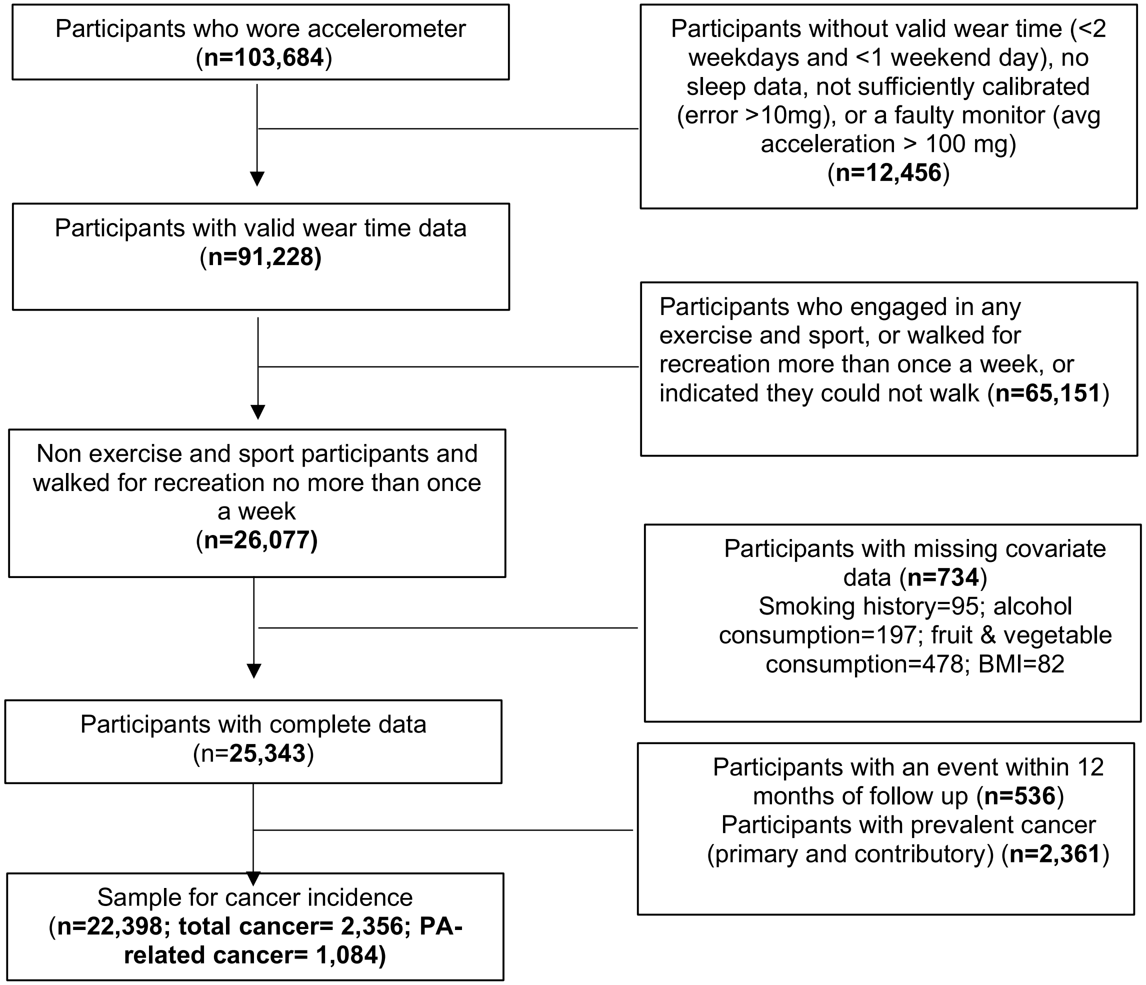

© 2023 Stamatakis E et al. *JAMA Oncol*

eFigure 2: Adjusted absolute risk dose-response of VILPA with total incident cancer

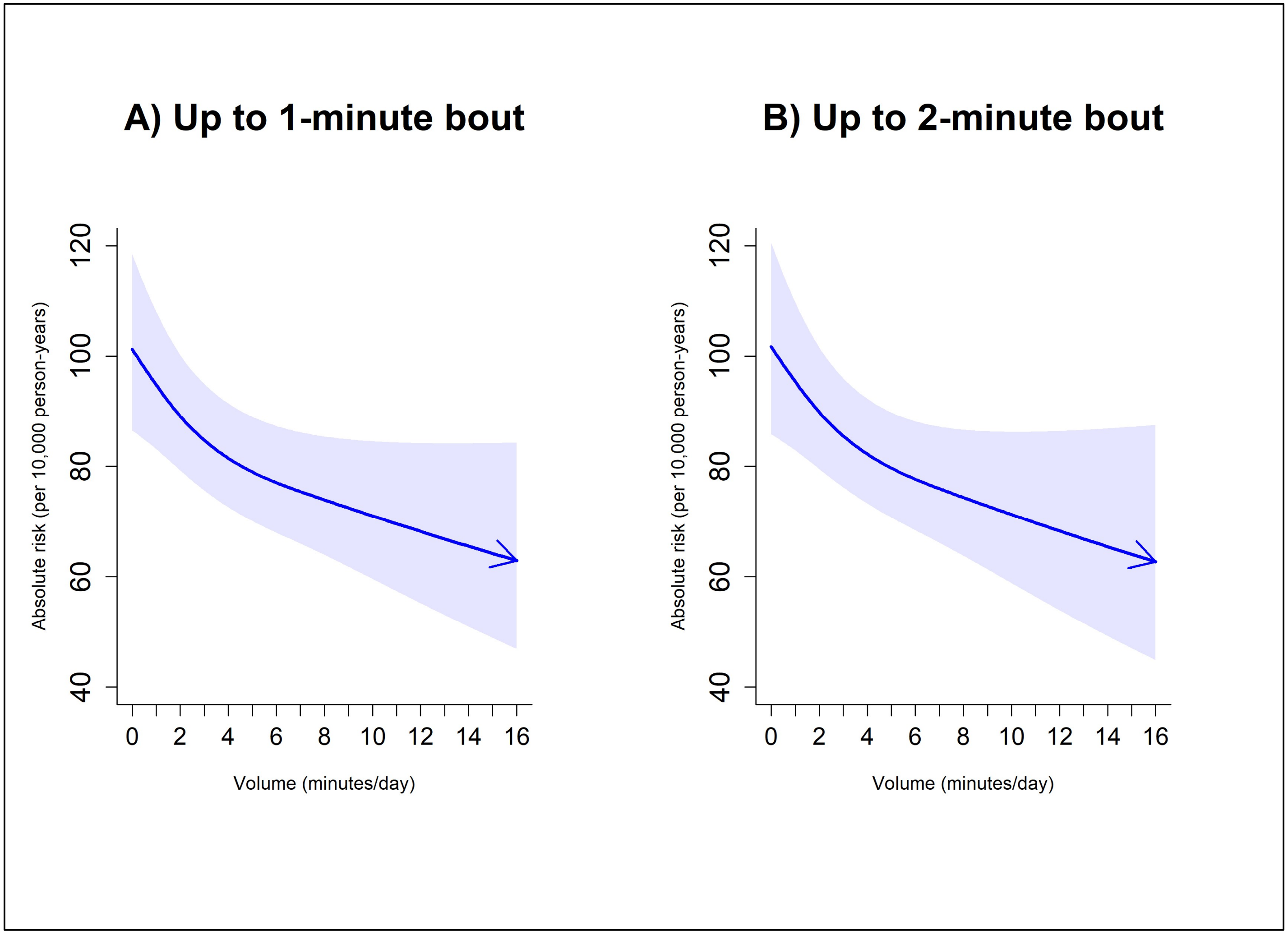

VILPA: vigorous intermittent lifestyle physical activity. Absolute risk adjusted for age, sex, body mass index, duration of light intensity physical activity, duration of moderate intensity physical activity, smoking history, alcohol consumption, accelerometer estimated sleep duration, fruit and vegetable consumption, education, medication use, self-reported parental history of cancer, and prevalent CVD. All analyses were additionally adjusted for vigorous physical activity duration lasting more than two minutes.

© 2023 Stamatakis E et al. *JAMA Oncol*

eFigure 3: Dose-response of up to 1-minute VILPA duration with exclusion for poor health, underweight, and cancer events occurring during the first 2 years of follow up (n=21,011)

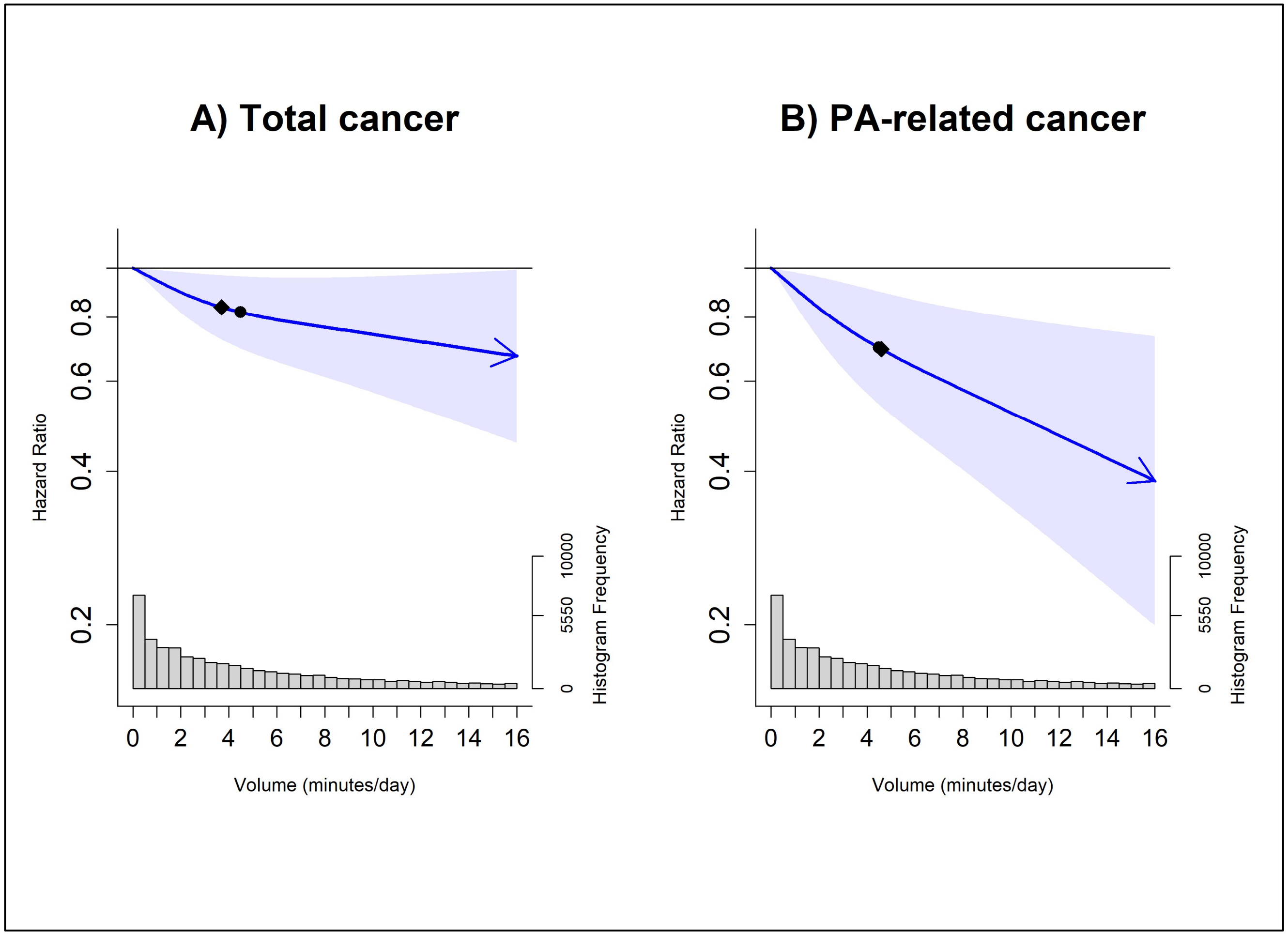

Diamond: ED50 value, the minimal dose, defined as the duration of VILPA associated with 50% of the optimal risk reduction; Circle: Median VILPA value. Analyses adjusted for age, sex, body mass index, duration of light intensity physical activity, duration of moderate intensity physical activity, smoking history, alcohol consumption, accelerometer estimated sleep duration, fruit and vegetable consumption, education, medication use, self-reported parental history of cancer, and prevalent CVD. All analyses were additionally adjusted for vigorous physical activity duration lasting more than two minutes. Hazard ratios calculated from Fine-Gray models

© 2023 Stamatakis E et al. *JAMA Oncol*

eFigure 4: Dose-response of up to 1-minute VILPA duration with no adjustment for body mass index (n= 22,507)

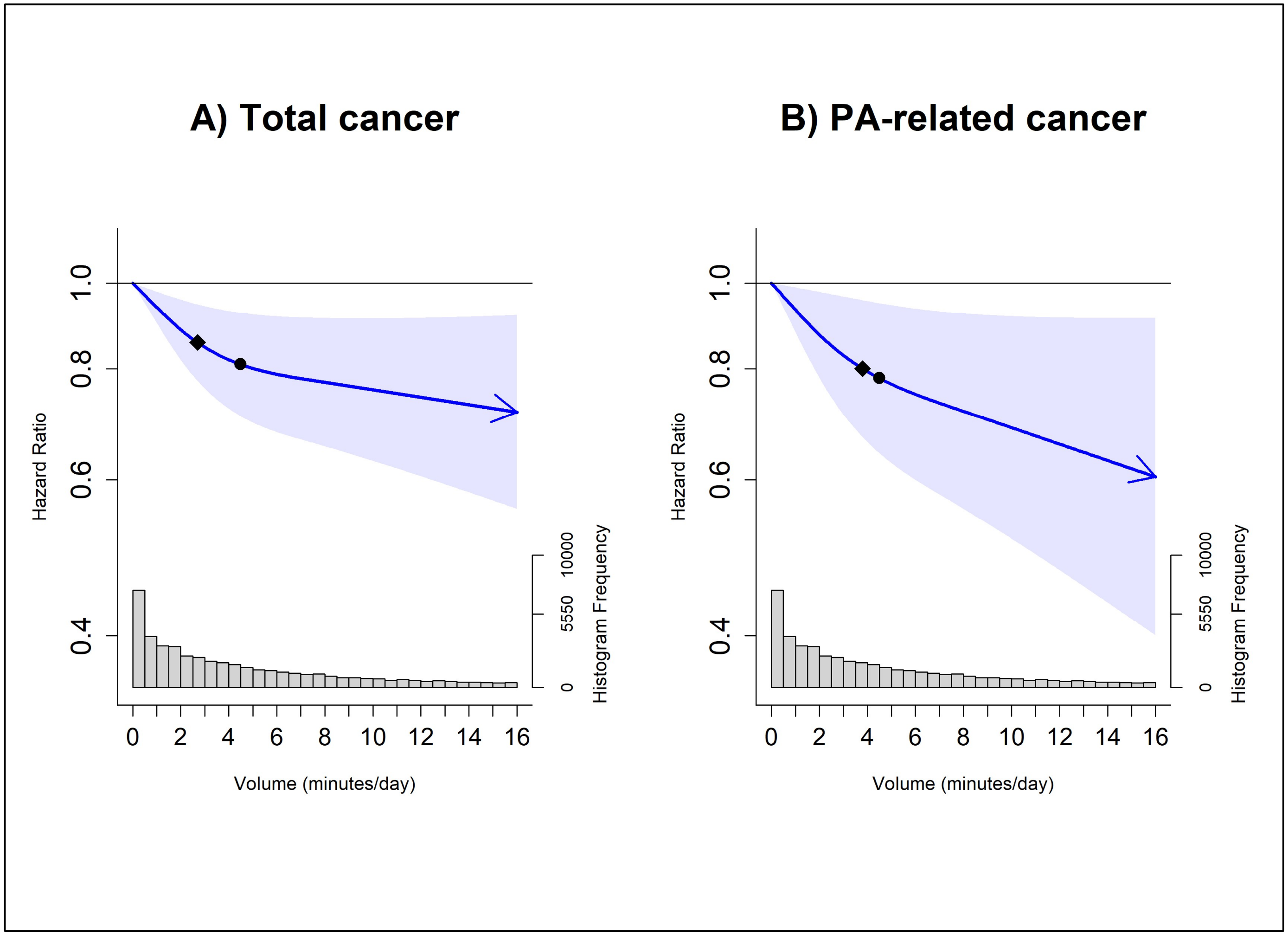

Diamond: ED50 value, the minimal dose, defined as the duration of VILPA associated with 50% of the optimal risk reduction; Circle: Median VILPA value. Analyses adjusted for age, sex, duration of light intensity physical activity, duration of moderate intensity physical activity, smoking history, alcohol consumption, accelerometer estimated sleep duration, fruit and vegetable consumption, education, medication use, self-reported parental history of cancer, and prevalent CVD. All analyses were additionally adjusted for vigorous physical activity duration lasting more than two minutes. Hazard ratios calculated from Fine-Gray models

© 2023 Stamatakis E et al. *JAMA Oncol*

eFigure 5: Dose-response of up to 1-minute VILPA duration with adjustment for VILPA energy expenditure (KJ/kg per day)

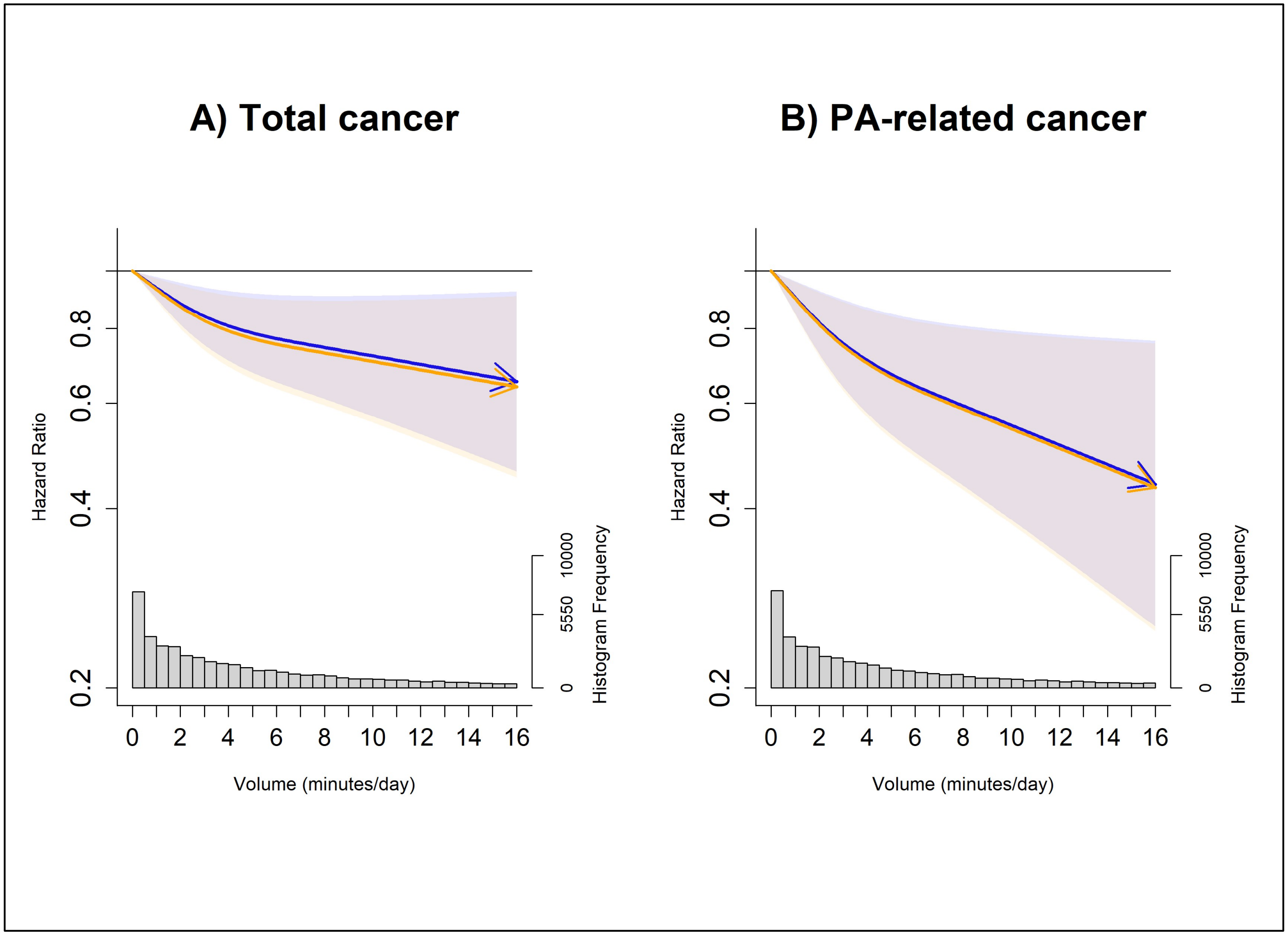

Blue= main analysis; Orange= main analysis with adjustment for VILPA energy expenditure (KJ/kg per day). Analyses adjusted for age, sex, duration of light intensity physical activity, duration of moderate intensity physical activity, smoking history, alcohol consumption, accelerometer estimated sleep duration, fruit and vegetable consumption, education, medication use, self-reported parental history of cancer, and prevalent CVD. All analyses were additionally adjusted for vigorous physical activity duration lasting more than two minutes. Adjustment for energy expenditure was done with the residual method Hazard ratios calculated from Fine-Gray models

© 2023 Stamatakis E et al. *JAMA Oncol*

eFigure 6: Sequential dose-response of up to 1-minute VILPA duration

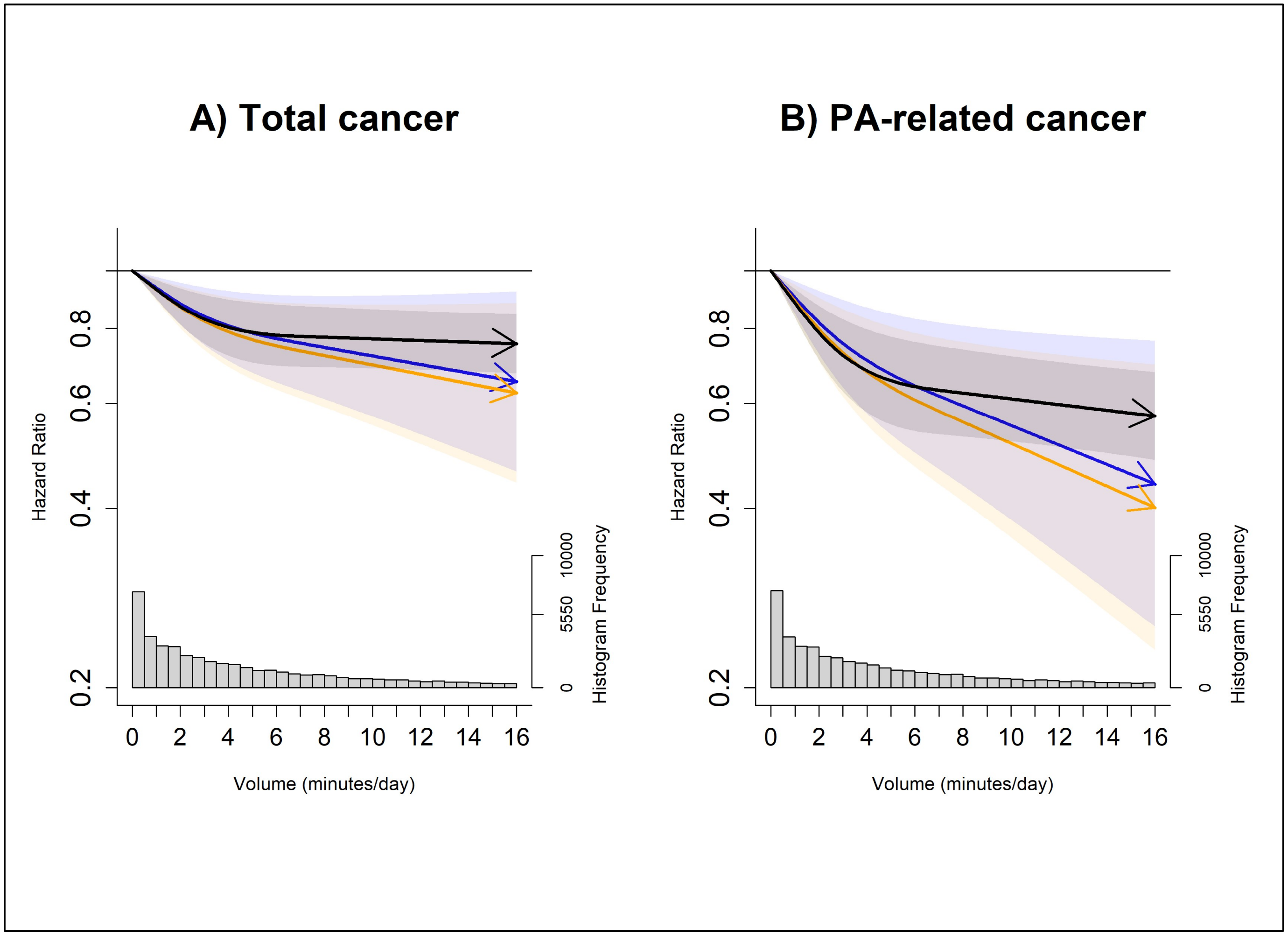

Black= Model 1 (age and sex adjustment); Orange= Model 2 (Model 1 plus light intensity activity, moderate intensity, BMI, smoking status, alcohol consumption, sleep duration, diet); Blue= Main analysis. Analyses adjusted for age, sex, duration of light intensity physical activity, duration of moderate intensity physical activity, smoking history, alcohol consumption, accelerometer estimated sleep duration, fruit and vegetable consumption, education, medication use, self-reported parental history of cancer, and prevalent CVD. All analyses were additionally adjusted for vigorous physical activity duration lasting more than one minutes. Adjustment for energy expenditure was done with the residual method Hazard ratios calculated from Fine-Gray models

© 2023 Stamatakis E et al. *JAMA Oncol*

eFigure 7: Categorical analysis of VILPA with total cancer and PA-related cancer

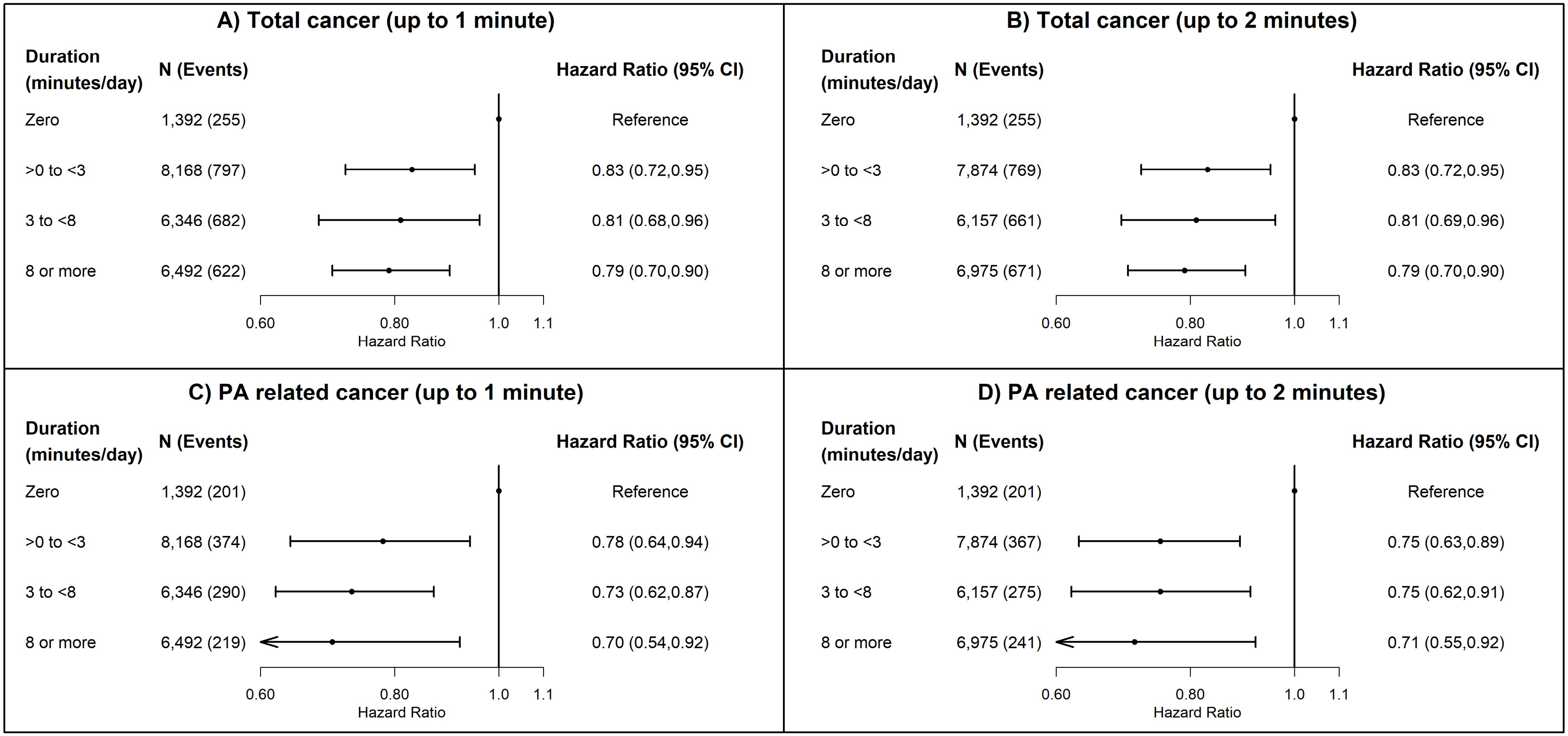

Analyses adjusted for age, sex, body mass index, duration of light intensity physical activity, duration of moderate intensity physical activity, smoking history, alcohol consumption, accelerometer estimated sleep duration, fruit and vegetable consumption, education, medication use, self-reported parental history of cancer, and prevalent CVD. All analyses were additionally adjusted for vigorous physical activity duration lasting more than one (bouts up to 1 minute exposure) or more than two (bouts up to 2 minutes exposure) minutes. Hazard ratios calculated from Fine-Gray models

© 2023 Stamatakis E et al. *JAMA Oncol*

eFigure 8: Dose-response association of Vigorous Intermittent Lifestyle Physical Activity (VILPA) daily duration from bouts lasting up to one and two minutes with total cancer incidence; adjustment for smoking pack-years (n=19,103; events=1,962). For direct comparisons with the main analyses we also present results in the same sample of the analyses adjusted for the existing smoking status variable.

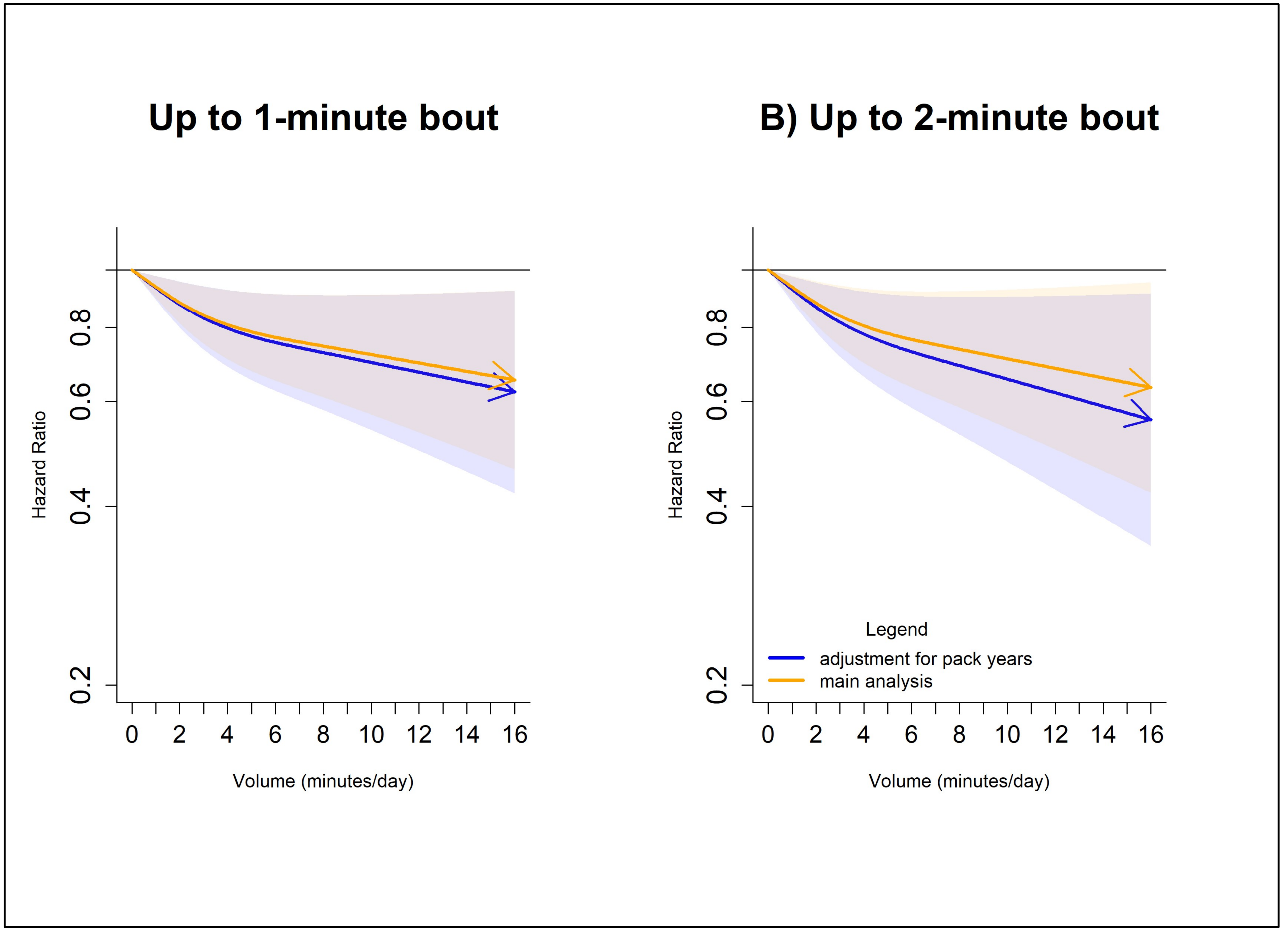

Analyses adjusted for age, sex, body mass index, duration of light intensity physical activity, duration of moderate intensity physical activity, smoking history (pack years), alcohol consumption, accelerometer estimated sleep duration, fruit and vegetable consumption, education, medication use, self-reported parental history of cancer, and prevalent CVD. All analyses were additionally adjusted for vigorous physical activity duration lasting more than one (bouts up to 1 minute exposure) or more than two (bouts up to 2 minutes exposure) minutes. Hazard ratios calculated from Fine-Gray models

© 2023 Stamatakis E et al. *JAMA Oncol*

eFigure 9: Dose-response association of Vigorous Intermittent Lifestyle Physical Activity (VILPA) daily duration from bouts lasting up to one and two minutes with PA-related cancer incidence; adjustment for smoking pack-years (n=19,103; events=913). For direct comparisons with the main analyses we also present results in the same sample of the analyses adjusted for the existing smoking status variable

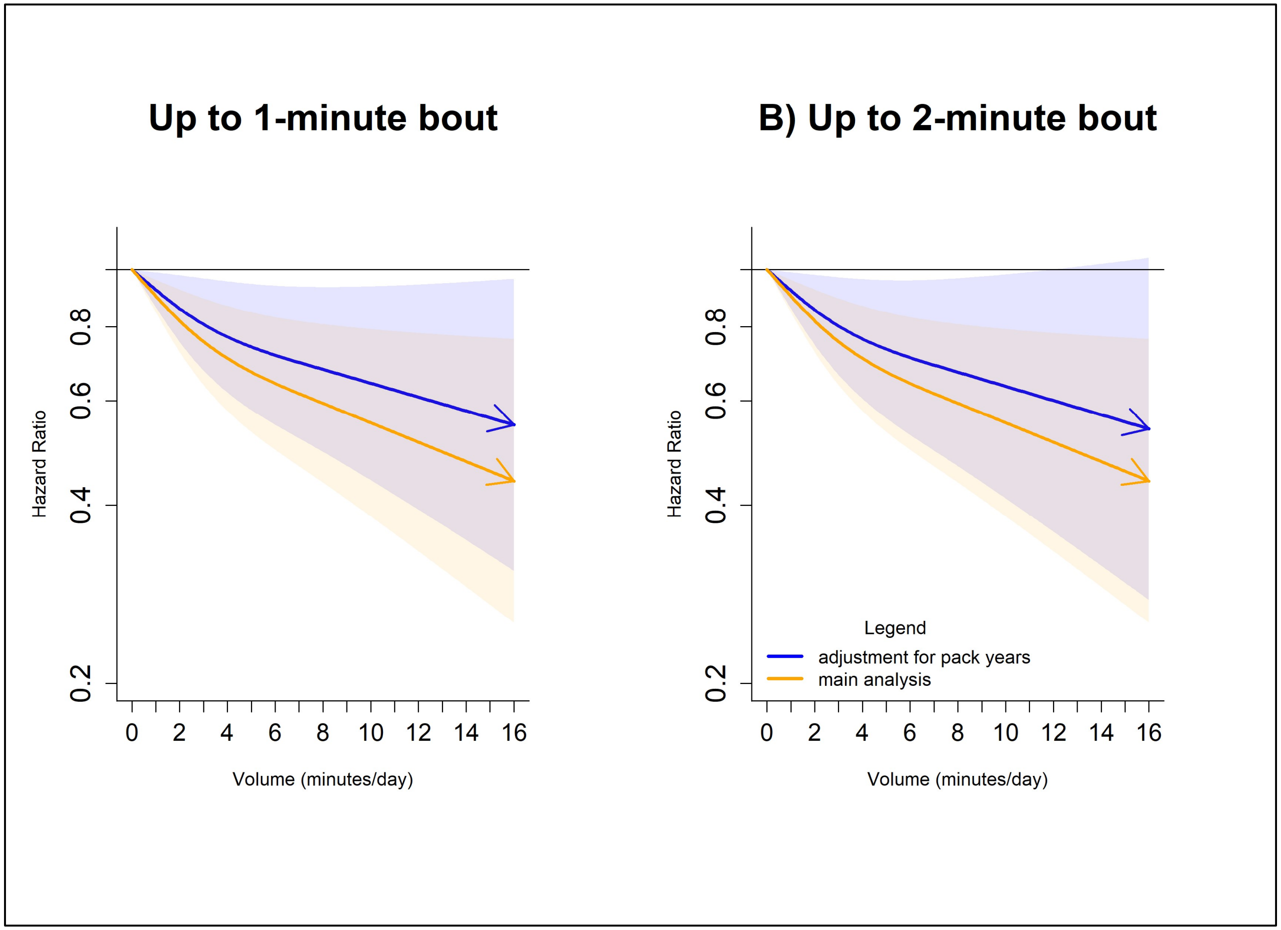

Analyses adjusted for age, sex, body mass index, duration of light intensity physical activity, duration of moderate intensity physical activity, smoking history (pack years), alcohol consumption, accelerometer estimated sleep duration, fruit and vegetable consumption, education, medication use, self-reported parental history of cancer, and prevalent CVD. All analyses were additionally adjusted for vigorous physical activity duration lasting more than one (bouts up to 1 minute exposure) or more than two (bouts up to 2 minutes exposure) minutes. Hazard ratios calculated from Fine-Gray models

© 2023 Stamatakis E et al. *JAMA Oncol*

eFigure 10: Dose-response association of Vigorous Intermittent Lifestyle Physical Activity (VILPA) daily duration from bouts lasting up to one and two minutes with total cancer incidence; adjustment for weekly units of alcohol (See **eTable 4**).

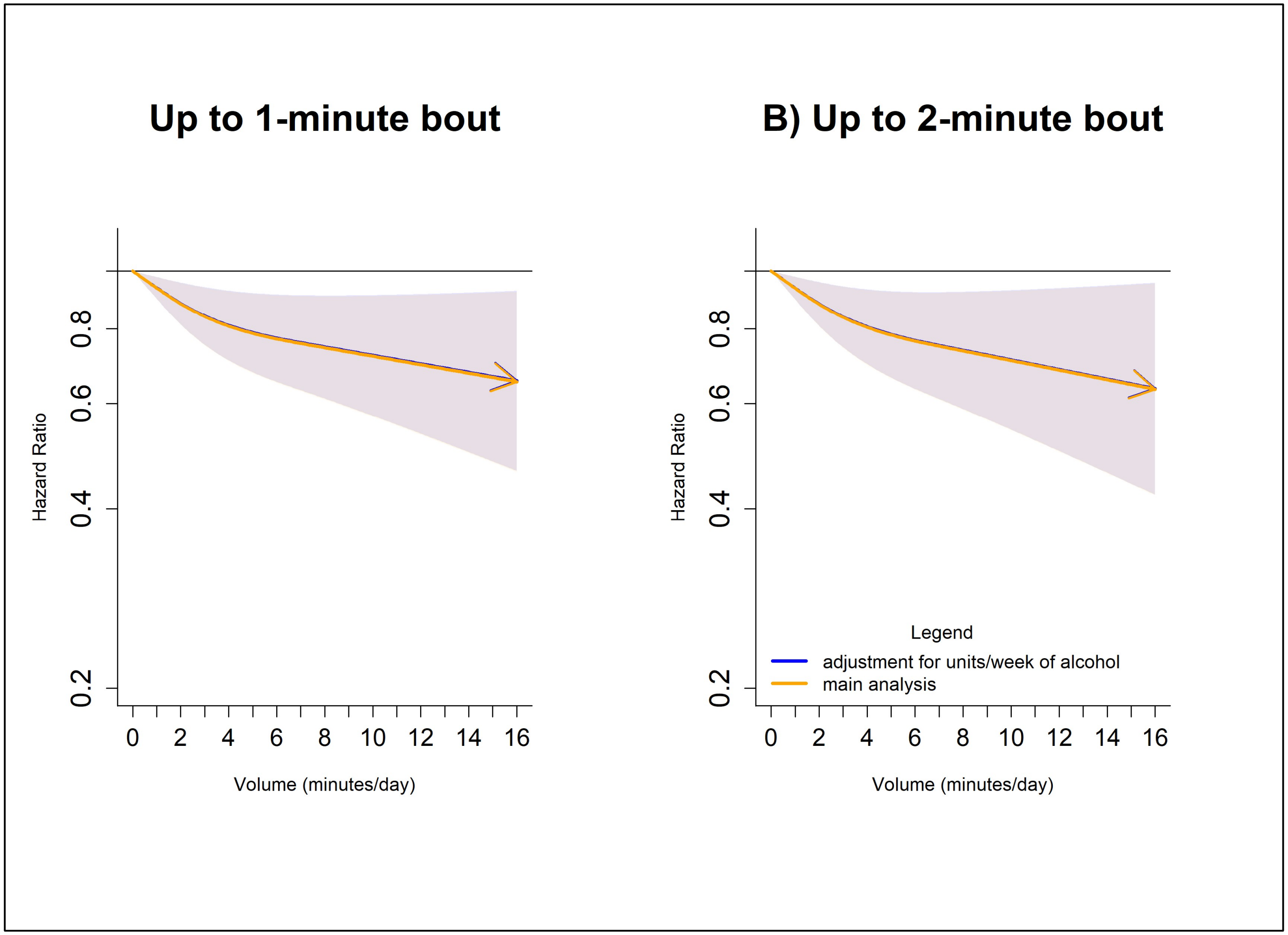

Analyses adjusted for age, sex, body mass index, duration of light intensity physical activity, duration of moderate intensity physical activity, smoking history, alcohol consumption (Never drinker, Ex-drinker, Within guidelines and <1 unit/wk, Within guidelines and 1 to 4 units/wk, Within guidelines and >4 units/wk, Above guidelines and <15 units/wk, Above guidelines and ≥15 units/wk), accelerometer estimated sleep duration, fruit and vegetable consumption, education, medication use, self-reported parental history of cancer, and prevalent CVD. All analyses were additionally adjusted for vigorous physical activity duration lasting more than one (bouts up to 1 minute exposure) or more than two (bouts up to 2 minutes exposure) minutes. Hazard ratios calculated from Fine-Gray models

© 2023 Stamatakis E et al. *JAMA Oncol*

eFigure 11: Dose-response association of Vigorous Intermittent Lifestyle Physical Activity (VILPA) daily duration from bouts lasting up to one and two minutes with PA-related incidence; adjustment for units per day of alcohol.

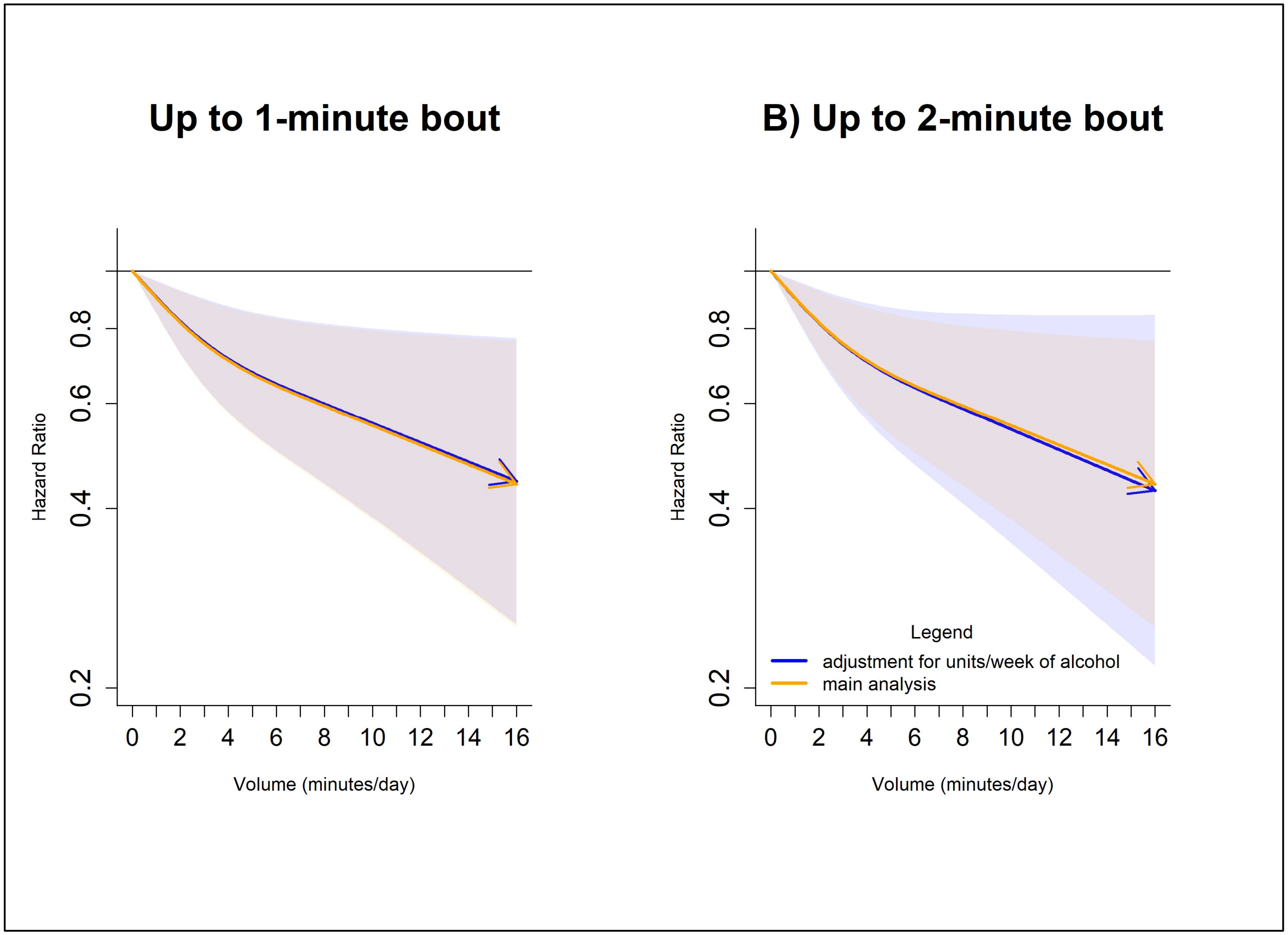

Analyses adjusted for age, sex, body mass index, duration of light intensity physical activity, duration of moderate intensity physical activity, smoking history, alcohol consumption (Never drinker, Ex-drinker, Within guidelines and <1 unit/wk, Within guidelines and 1 to 4 units/wk, Within guidelines and >4 units/wk, Above guidelines and <15 units/wk, Above guidelines and ≥15 units/wk), accelerometer estimated sleep duration, fruit and vegetable consumption, education, medication use, self-reported parental history of cancer, and prevalent CVD. All analyses were additionally adjusted for vigorous physical activity duration lasting more than one (bouts up to 1 minute exposure) or more than two (bouts up to 2 minutes exposure) minutes. Hazard ratios calculated from Fine-Gray models

© 2023 Stamatakis E et al. *JAMA Oncol*

eFigure 12: Dose-response association of Vigorous Intermittent Lifestyle Physical Activity (VILPA) daily duration from bouts lasting up to one and two minutes with total cancer incidence; with adjustment for prevalent diabetes.

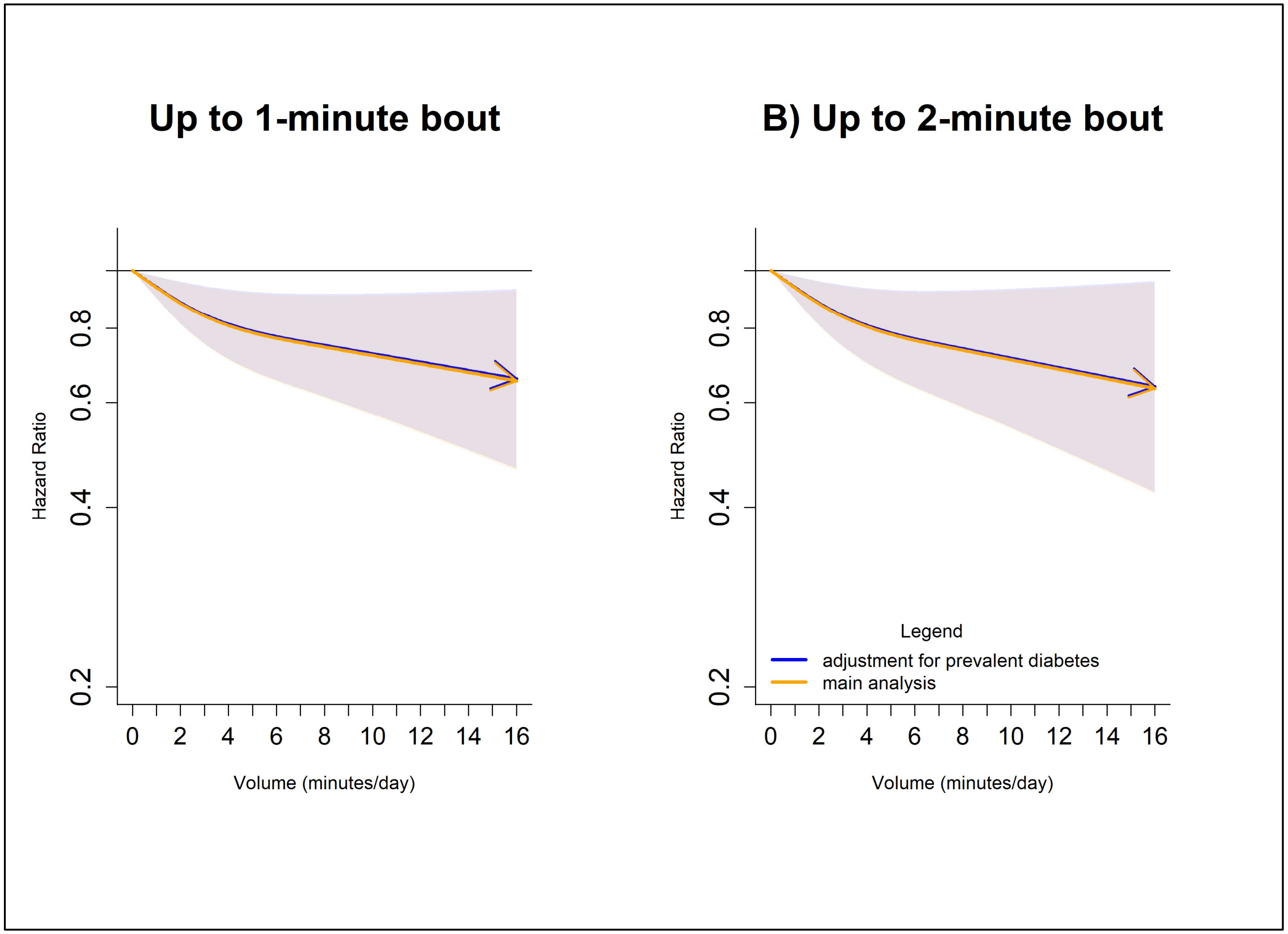

Analyses adjusted for age, sex, body mass index, duration of light intensity physical activity, duration of moderate intensity physical activity, smoking history, alcohol consumption, prevalent diabetes, accelerometer estimated sleep duration, fruit and vegetable consumption, education, medication use, self-reported parental history of cancer, and prevalent CVD. All analyses were additionally adjusted for vigorous physical activity duration lasting more than one (bouts up to 1 minute exposure) or more than two (bouts up to 2 minutes exposure) minutes. Hazard ratios calculated from Fine-Gray models

© 2023 Stamatakis E et al. *JAMA Oncol*

eFigure 13: Dose-response association of Vigorous Intermittent Lifestyle Physical Activity (VILPA) daily duration from bouts lasting up to one and two minutes with PA-related incidence; adjustment for prevalent diabetes.

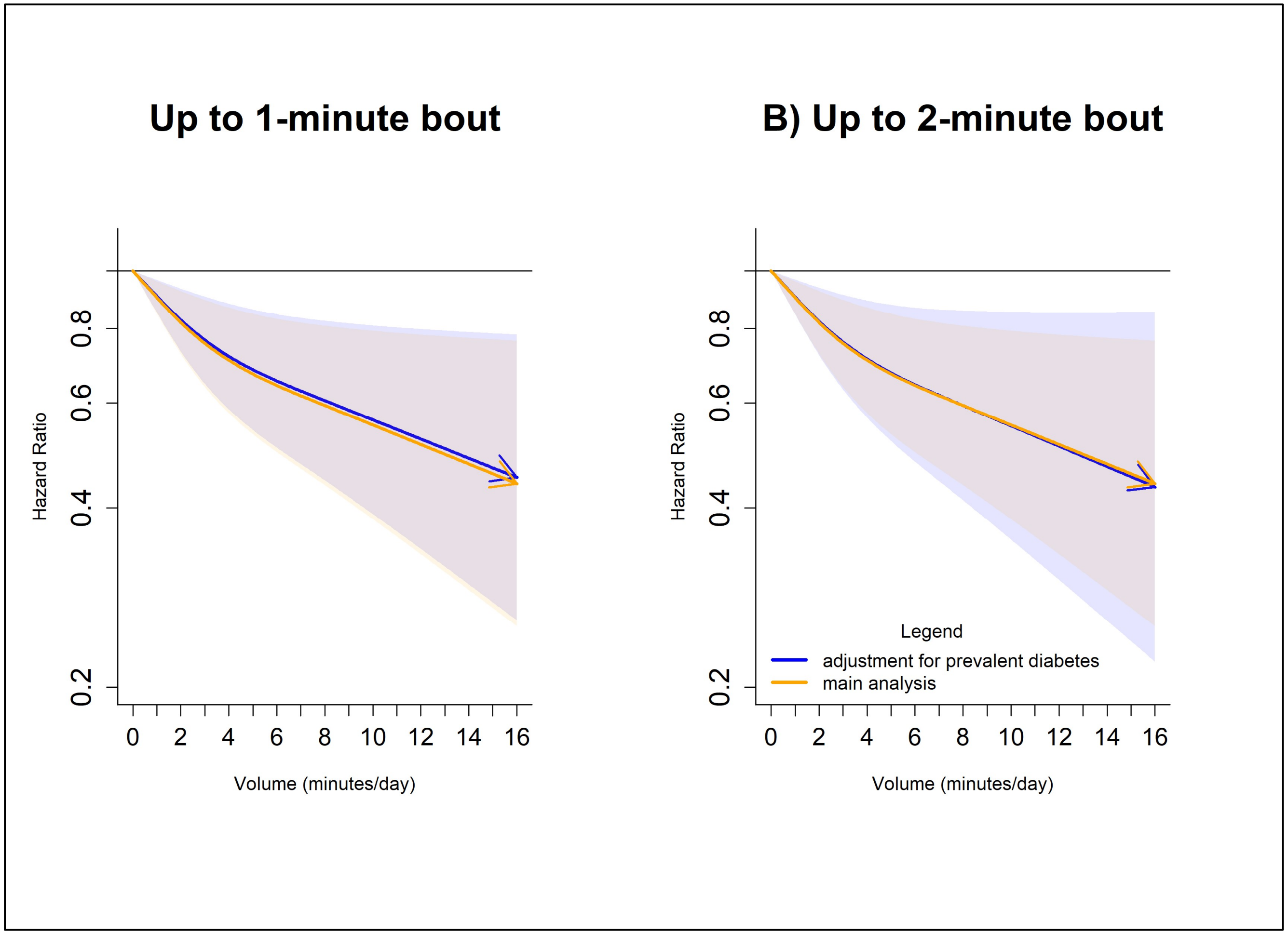

Analyses adjusted for age, sex, body mass index, duration of light intensity physical activity, duration of moderate intensity physical activity, smoking history, alcohol consumption, prevalent diabetes, accelerometer estimated sleep duration, fruit and vegetable consumption, education, medication use, self-reported parental history of cancer, and prevalent CVD. All analyses were additionally adjusted for vigorous physical activity duration lasting more than one (bouts up to 1 minute exposure) or more than two (bouts up to 2 minutes exposure) minutes. Hazard ratios calculated from Fine-Gray models

© 2023 Stamatakis E et al. *JAMA Oncol*

eMethods: unabridged sampling, design, and physical activity-related methods

Follow-up ascertainment descriptives

In our analytic sample of 22,398 participants, 2,574 wore the accelerometer in 2013, 10,051 wore the accelerometer in 2014, and 9,773 wore the accelerometer in 2015. 20,255 were from England, 1,286 were from Scotland, and 857 were from Wales.

Deaths were ascertained through linkage with the National Health Service Digital of England and Wales or the National Health Service Central Register and National Records of Scotland. Censoring for England and Wales was up to September 30th, 2021. Censoring for Scotland was up to October 31st, 2021.

Hospital inpatient data was ascertained through linkage with the National Health Service Digital for England, Information and Statistics Division for Scotland, and Secure Anonymised Information Linkage for Wales. Censoring for England and Scotland was up to September 30th, 2021 and July 31st, 2021, respectively. Censoring for Wales was up to February 28th, 2018.

Cancer registry data was ascertained through linkage with the National Health Service Digital for England and Wales, and the National Records of Scotland and National Health Service Central Register for Scotland. Censoring for England and Wales was up to February 29th, 2020. Censoring for Scotland was up to January 31st, 2021.

Sample & design

The UK Biobank Study is a prospective cohort study of adults aged between 40-69 years whose baseline measurements took place between 2006-10. Participants provided informed consent and ethical approval was provided by the UK's National Health Service, National Research Ethics Service (Ref80 11/NW/0382).

Between 2013 and 2015 (median 5.5 years after the baseline measurements), 103,684 UK Biobank participants wore a wrist-worn accelerometer for 7 days[1,2]. We excluded participants with missing covariates and insufficient valid wear days. Monitoring days were considered valid if wear time was greater than 16 hours. To be included in analysis, participants were required to have at least three valid monitoring days, with at least one of those days being a weekend day. We excluded participants who reported that they cannot walk.

To enable examination of VILPA in our study (i.e. brief bouts of non-exercise vigorous physical activity occurring during daily living), we only included participants who reported no leisure time exercise participation and no more than one recreational walk per week.

© 2023 Stamatakis E et al. *JAMA Oncol*

Participation in exercise and recreational walking was measured through a close-ended touch-screen questionnaire that asked participants to report if, how often, and for how long they participate in such activities **(Supplementary Table 1)**. Among the included 14,982 participants who were walking for recreation once a week or less, the average spacing of VILPA bouts was 165.7 (47.0) minutes within days and 16.7 (5.5) hours between days (last session of a day vs first session the day after). The modal median length of the (at most) one and only weekly walking session these participants reported was 30-60 minutes (32.5% of the 14,982 participants), effectively eliminating the possibility that the device-recorded VILPA bouts occurred during recreational walking.

To provide a comparison between effects of VILPA and (context-agnostic) VPA we repeated the main analyses among "exercisers", defined as those UK Biobank accelerometry sub-study participants who did not meet the above criteria to be considered non-exercisers, i.e., those who reported any leisure time exercise or more than one recreational walking session per week **(Supplementary Table 1)**.

Definition of VILPA & choice of bout length

We based the choice of VILPA bouts length entered in our analyses on an ongoing study of 58 adults (mean age 55.7 (SD=10.1) years) aimed at developing an empirical definition of VILPA (unpublished data). Participants completed five activities of daily living while wearing an indirect calorimetry unit (Cosmed K5) and Polar heart-rate monitor. The activities included: 1) Walking on a flat surface at a self-selected "very fast" pace; 2) Walking on a flat surface whilst carrying shopping-like bags equivalent to 5% of body weight at a self-defined "fast" pace; 3) Walking on a flat surface whilst carrying shopping-like bags equivalent to 10% of body weight at a self-defined "fast" pace; 4) walking at 2.5% gradient at a self-defined "very fast" pace (treadmill); 5) walking at 7.0% gradient at a self-defined "very fast" pace (treadmill). The sequence of activities was randomised for each participant and counterbalanced across participants to prevent biases due to residual fatigue accumulating during the protocol.

Participants performed each activity until vigorous intensity was reached for two out of three criteria: 1) %VO2max (≥64%); 2) %HRmax (≥77%); 3) Rating of perceived exertion (Borg Scale) ≥15. For %VO2max and %HRmax, the threshold had to be met for at least 30 consecutive seconds to minimise the effects of noise. VO_2max was calculated using the Ebbeling treadmill test and heart rate max was calculated using the Tanaka equation[3]. Between activities, participants had 5 minutes of seated recovery, or until heart rate and breathing returned to resting levels. Resting VO_2 and heart rate were measured at the beginning of each session with the participant lying supine using 5 minutes of steady-state (%CV ≤10%). The duration to reach vigorous intensity across all five activities is shown in **Supplemental Text 1 Display Item A** below. As the mean time required to reach vigorous intensity in two of the above three physiological intensity indices was 73.5(SD=26.2) seconds across all activities, we decided to test VILPA bouts lasting up to one and up to two minutes in the present analyses.

Display Item A: Mean durations to reach vigorous intensity across five activities (unpublished data)

	$\%HR_{max}$	$\%VO_{2max}$	Rating of perceived exertion
Walking at a fast pace on a flat surface	76.2 (22.9) sec.	71.5 (18.9) sec	53.6 (19.7) sec
Walking at a fast pace carrying 5% of body weight	64.4 (32.4) sec	60.8 (25.2) sec	44.1 (23.7) sec
Walking at a fast pace carrying 10% of body weight	73.1 (34.6) sec	68.8 (21.3) sec	40.1 (20.3) sec
Walking at a fast pace on a 2.5% incline	85.4 (20.3) sec	83.0 (20.2) sec	66.8 (21.3) sec
Walking at a fast pace on a 7.0% incline	80.3 (28.0) sec	75.7 (15.8) sec	63.6 (21.9) sec

© 2023 Stamatakis E et al. *JAMA Oncol*

Wearable device-based physical activity classification

eMethods 1 Display Item B below summarises how activity intensity was classified using a previously validated Random Forest (RF) activity classifier[4]. RF is an ensemble of multiple decision trees. Each tree is learned on a bootstrap sample of training data and each node in the tree is split using the best among a randomly selected set of acceleration features. The decisions from each tree are aggregated and a final model prediction is based on majority vote. The RF model requires very little pre-processing of the data, as the features do not need to be normalized. Additionally, the model is resistant to over fitting the training data because each tree within the forest is independently grown to maximum depth using a randomly selected subset of features.

Display Item B: Physical activity type and intensity diagram

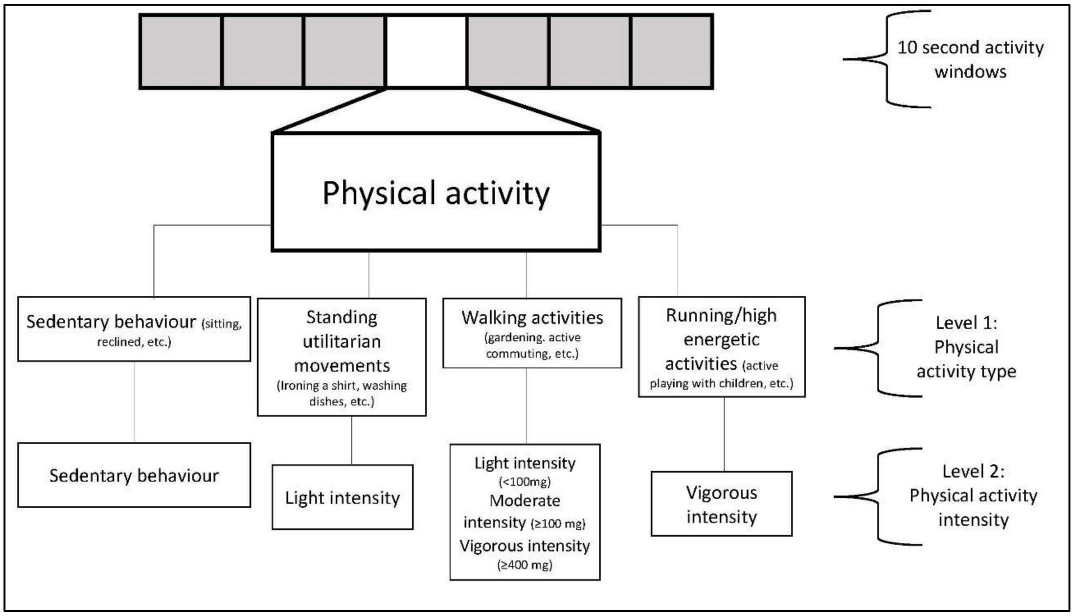

This 2-stage classifier which first categorized physical activity in 10 second windows into one of ,four activity classes: sedentary, standing utilitarian movements (e.g., ironing a shirt, washing dishes), walking activities (e.g. gardening, active commuting, mopping floors), running/high energetic activities (e.g. active playing with children). These activity classes were then assigned to one of four activity intensities: sedentary, light, moderate, and vigorous. Walking activities were classified as light (an acceleration value of <100mg), moderate (≥=100mg) and vigorous (≥=400mg) intensity[5]. For example, a VILPA bout lasting up to 2 minutes, 12 consecutive 10-second windows needed to be classified as vigorous. When there were more than 12 vigorous consecutive activity windows these bouts counted as long vigorous physical activity sessions in the corresponding analyses (2.3% of all vigorous physical activity bouts). Differentiation between sleep[6] and non-wear[7] was identified using the change in tilt angle and acceleration standard deviation. Monitors were calibrated[8] and corrected for orientation[9] using previously published methods, although residual signal and alignment uncertainties may persist.

© 2023 Stamatakis E et al. *JAMA Oncol*

Activities in an independent sample of 98 participants (Age = 56.4 ± 15.7; 53.1% female) from the US[10] (University of California Irvine Center for Machine Learning and Intelligent Systems *Physical Activity Monitoring for Aging People* study [published data], accessible at https://archive.ics.uci.edu/ml/datasets) and Australia[11] (University of Queensland *Where and When at Work* study [published data], and University of Sydney *Intermittent Lifestyle Physical Activity* Study [unpublished data]) providing 103,607 activity samples from structured and free-living activities (17,267 minutes) were used to assess robustness and generalizability of the classifier (S**upplemental Text 1 Display Item C and D**). For free-living activities participant-worn or researcher-held Go-Pro video-recordings were used to attain ground-truth physical activity. Video files were imported into the Noldus Observer XT software for continuous direct observation coding. A two-stage direct observation scheme was implemented in which the participant's movement behaviour was coded for activity type and then activity intensity based on Compendium of Physical Activities[12]. The direct observation system generated a vector of date-time stamps corresponding to the start and finish of each movement event, which were used to assign the activity codes to the corresponding time segments of the accelerometer data. Interobserver reliability was assessed by dual coding. The intraclass correlation coefficient for coding activities was 0.91 (0.87-0.94).

Display Item C: Intensity classification performance in 98 US and Australian adults

	Sensitivity	Specificity	Precision	F-score	Overall Accuracy	Weighted Kappa	Overall F-score
Sedentary	86.5	93.7	90.5	88.5			
Light	71.2	89.4	55.8	62.6			
Moderate	85.4	96.6	92.7	88.9			
Vigorous	95.4	99.4	94.6	95.0			
					84.6	0.78	83.8

Display Item D: Confusion matrix of activity classification in 98 US and Australian adults

	Sedentary	Light	Moderate	Vigorous
Sedentary	**36,904**	5,232	508	2
Light	3,120	**11,712**	1,612	17
Moderate	502	4,016	**29,528**	526
Vigorous	226	17	214	**9,470**

Rows= ground truth; columns=predictions; bold=correct labels; numbers represent each 10-second window; Derived from the US and Australian datasets

Performance was further evaluated in a separate sample of 151 adults (age range 18-91 years, 65.6% female; **Supplemental Text 1 Display Item E** recruited from the UK[13] (University of Oxford *Capture 24* study [published data], accessible at

© 2023 Stamatakis E et al. *JAMA Oncol*

https://ora.ox.ac.uk/objects/uuid:99d7c092-d865-4a19-b096-cc16440cd001). Participants in this dataset wore body cameras that provided pictures every 20 seconds to annotate ground-truth free-living activity labels. The picture-based activity coding scheme has been previously described[9]. A total of 172,360 activity samples (28,727 minutes) were provided by participants.

Display Item E: Participant-level specific recall and precision of activity classification in 151 UK adults

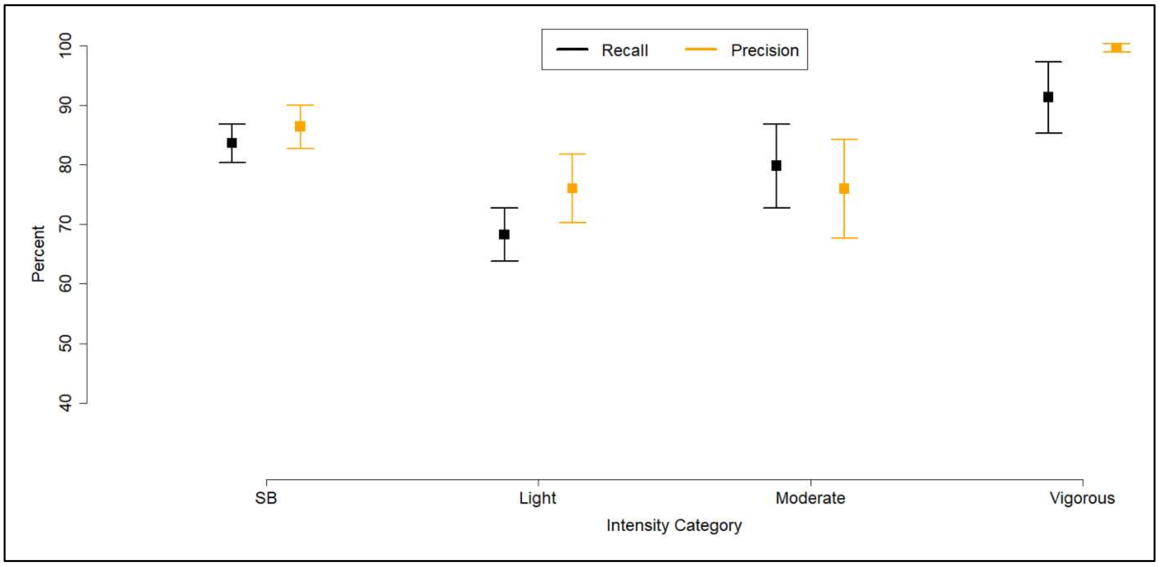

© 2023 Stamatakis E et al. *JAMA Oncol*

REFERENCES

1. Ramakrishnan R, Doherty A, Smith-Byrne K, et al. Accelerometer measured physical activity and the incidence of cardiovascular disease: Evidence from the UK Biobank cohort study. *PLoS medicine.* 2021;18(1):e1003487.
2. Doherty A, Jackson D, Hammerla N, et al. Large scale population assessment of physical activity using wrist worn accelerometers: The UK Biobank Study. *PloS one.* 2017;12(2):e0169649.
3. Tanaka H, Monahan KD, Seals DR. Age-predicted maximal heart rate revisited. *J Am Coll Cardiol.* 2001;37(1):153-156.
4. Pavey TG, Gilson ND, Gomersall SR, Clark B, Trost SG. Field evaluation of a random forest activity classifier for wrist-worn accelerometer data. *Journal of science and medicine in sport.* 2017;20(1):75-80.
5. Hildebrand M, VT VANH, Hansen BH, Ekelund U. Age group comparability of raw accelerometer output from wrist- and hip-worn monitors. *Med Sci Sports Exerc.* 2014;46(9):1816-1824.
6. van Hees VT, Sabia S, Jones SE, et al. Estimating sleep parameters using an accelerometer without sleep diary. *Sci Rep.* 2018;8(1):12975.
7. Ahmadi MN, Nathan N, Sutherland R, Wolfenden L, Trost SG. Non-wear or sleep? Evaluation of five non-wear detection algorithms for raw accelerometer data. *Journal of sports sciences.* 2020;38(4):399-404.
8. Sipos M, Paces P, Rohac J, Novacek P. Analyses of triaxial accelerometer calibration algorithms. *IEEE Sensors Journal.* 2011;12(5):1157-1165.
9. Mizell D. Using gravity to estimate accelerometer orientation. Paper presented at: Seventh IEEE International Symposium on Wearable Computers, 2003. Proceedings.2003.
10. Reiss A, Weber M, Stricker D. Exploring and extending the boundaries of physical activity recognition. Paper presented at: 2011 IEEE International Conference on Systems, Man, and Cybernetics2011.
11. Clark BK, Winkler EA, Brakenridge CL, Trost SG, Healy GN. Using Bluetooth proximity sensing to determine where office workers spend time at work. *PloS one.* 2018;13(3):e0193971.
12. Ainsworth B, Haskell W, Herrmann S, et al. Second update of codes and MET values. *Med Sci Sports Exerc.* 2011;39:1575-1581.
13. Willetts M, Hollowell S, Aslett L, Holmes C, Doherty A. Statistical machine learning of sleep and physical activity phenotypes from sensor data in 96,220 UK Biobank participants. *Sci Rep.* 2018;8(1):7961.

© 2023 Stamatakis E et al. *JAMA Oncol*
