# Supplementary material for: Vigorous Intermittent Lifestyle Physical Activity and Cancer Incidence Among Nonexercising Adults: The UK Biobank Accelerometry Study
Source: JAMA Oncol. 2023 Jul 27;9(9):1255–9. doi: 10.1001/jamaoncol.2023.1830 (PMC10375384; doi:10.1001/jamaoncol.2023.1830)
Supplement: Supplement 2. — Data Sharing Statement [file jamaoncol-e231830-s002.pdf]

Data Sharing Statement

Stamatakis. Vigorous Intermittent Lifestyle Physical Activity and Cancer Incidence Among Nonexercising Adults. *JAMA Oncol*. Published July 27, 2023.
doi:10.1001/jamaoncol.2023.1830

Data
Data available: Yes
Data types: Other (please specify)
Additional Information: The data that support the findings of this study are available from the UK Biobank but restrictions apply to the availability of these data, which were used under license for the current study, and so are not publicly available. Data are however available from the authors upon reasonable request and with permission of the UK Biobank.
How to access data: The data that support the findings of this study are available from the UK Biobank but restrictions apply to the availability of these data, which were used under license for the current study, and so are not publicly available. Data are however available from the authors upon reasonable request and with permission of the UK Biobank.
When available: With publication

Supporting Documents
Document types: Statistical/analytic code
How to access documents: https://zenodo.org/record/8068111
When available: With publication

Additional Information
Who can access the data: Anyone
Types of analyses: for any purpose
Mechanisms of data availability: without investigator support,
Any additional restrictions: The data that support the findings of this study are available from the UK Biobank but restrictions apply to the availability of these data, which were used under license for the current study, and so are not publicly available. Data are however available from the authors upon reasonable request and with permission of the UK Biobank.
